# Surface Plasmon Resonance (SPR) Sensor for Cancer Biomarker Detection

**DOI:** 10.3390/bios13030396

**Published:** 2023-03-17

**Authors:** Sreyashi Das, Ram Devireddy, Manas Ranjan Gartia

**Affiliations:** Department of Mechanical and Industrial Engineering, Louisiana State University, Baton Rouge, LA 70803, USA

**Keywords:** cancer, biomarker, biosensor, surface plasmon resonance, liquid biopsy

## Abstract

A biomarker is a physiological observable marker that acts as a stand-in and, in the best-case scenario, forecasts a clinically significant outcome. Diagnostic biomarkers are more convenient and cost-effective than directly measuring the ultimate clinical outcome. Cancer is among the most prominent global health problems and a major cause of morbidity and death globally. Therefore, cancer biomarker assays that are trustworthy, consistent, precise, and verified are desperately needed. Biomarker-based tumor detection holds a lot of promise for improving disease knowledge at the molecular scale and early detection and surveillance. In contrast to conventional approaches, surface plasmon resonance (SPR) allows for the quick and less invasive screening of a variety of circulating indicators, such as circulating tumor DNA (ctDNA), microRNA (miRNA), circulating tumor cells (CTCs), lipids, and proteins. With several advantages, the SPR technique is a particularly beneficial choice for the point-of-care identification of biomarkers. As a result, it enables the timely detection of tumor markers, which could be used to track cancer development and suppress the relapse of malignant tumors. This review emphasizes advancements in SPR biosensing technologies for cancer detection.

## 1. Introduction

A biomarker is a biological discovery that anticipates a clinically significant endpoint or interim result. Biomarkers can be applied for disease detection, characterization, diagnosis, and monitoring. Understanding the pathophysiological relationship between a marker and a diagnostic and therapeutic endpoint is necessary to appreciate the importance of a biomarker [1].

Cancer is a multi-step process involving genetic and epigenetic modifications that disrupt the cellular homeostasis between cellular growth and death. Cancer is a major illness that kills millions of individuals annually throughout the globe [2]. According to the International Agency for Research on Cancer, there are approximately 18.1 million cancer occurrences and 9.6 million cancer deaths per year [3]. Cancer is among the most serious global health problems that cause morbidity and mortality in developed countries. Perhaps there is a connection between molecular and tissue-level changes that fuel malignant anomalies throughout the tissue and major contributors to cancer development [4]. The conclusion is that analyzing the biomolecules implicated in the molecular pathogenesis of cancer could yield important clinical information, i.e., biomarkers, which are crucial in detecting whether cancer is suspected. Nucleic acids, carbohydrates, proteins, lipids, and metabolites are among these molecules. Biomarkers can be utilized for various purposes, including determining an individual’s risk of acquiring cancer, forecasting the chance that a specific medication will be effective for a particular patient, and tracking the progression to see if a therapy is effective. There is an immediate emergency for credible, robust, validated cancer markers to minimize cancer mortality and morbidity. Biomarkers not only identify cancer but also categorize it by stage and kind.

Rapid and accurate cancer detection can strengthen the efficacy of treatment therapies, resulting in higher ultimate survival rates [5]. Substantial expectations are heaped on biosensors, which are gaining clinical utility with time. In this context, distinct sensing strategies are available based on electrochemistry, colorimetry, chemiluminescence, fluorescence spectroscopy, and surface plasmon resonance (SPR) [6,7,8,9]. Among the above-mentioned strategies, SPR is one of the most often used techniques in the field of on-the-spot detection of cancer biomarkers due to its non-destructive nature, rapid and real-time evaluation of the intended biomarker with excellent selectivity, and reproducibility [10,11]. These characteristics render it an excellent approach for detecting potential markers in cancers [12,13]. SPR is an optical sensor technology that detects alterations in the localized refractive index to assess molecule binding at a metal surface. This surface-sensitive technique may also be used to study interactions between mounted biomolecules and analytes since the metal–aqueous contact depth is typically 200 nm [14]. The SPR method has been demonstrated to be a successful high-throughput detection tool for markers in clinical samples for early cancer diagnosis [15]. SPR biosensors provide several advantages over conventional cancer detection methods, including detecting cancer in situ in real time without needing labels and with higher sensitivity [16]. SPR-based biosensors have already been described for detecting antibodies (Abs), proteins, therapeutics, viruses, and nucleic acids in cancer patient specimens [16]. For instance, a graphene-based SPR sensor was devised to identify the folic acid protein (FAP) for early-stage cancer diagnosis. This sensor detects FAP at femtomolar levels, rendering it ideal for quantitative clinical research [15].

SPR imaging is a new technique that integrates the benefits of classic SPR with high-throughput abilities, enabling researchers to track the interactions of thousands of biological molecules at once. SPR imaging can be a valuable tool for many types of biological research, such as drug discovery, proteome analysis, antibody creation, and pathway explanation, especially when combined with protein arrays. 

Several studies have been conducted applying SPR techniques in cancer biomarker detection. Exosomal biomarkers and miRNAs have been detected using SPR biosensors [13,17]. According to a literature review, few initiatives have been made to use SPR to identify several potential biomarkers in distinct types of cancer. This study summarizes the current advancements in SPR approaches for sensing various potential cancer biomarkers and the role of SPR in cancer drug discovery and therapeutic antibody development. To aid in developing a framework for the creation of future SPR sensor systems, recent developments in the use of SPR sensors to measure trace levels of potential cancer biomarkers are also described. 

## 2. Biomarkers in Cancer Detection, Diagnosis, and Prognosis

**Figure 1:**
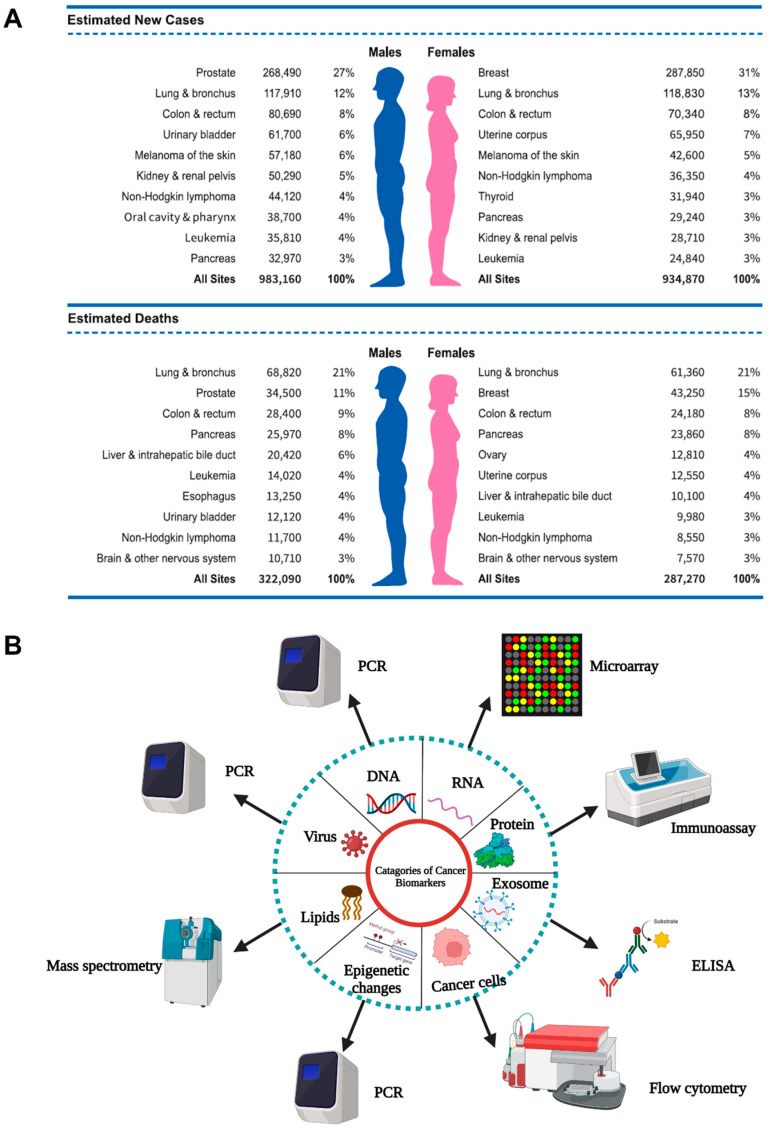
(**A**) Top ten major cancers and mortalities by sex in 2022 (USA) [25]. (**B**) Schematic representation of cancer biomarkers and their detection approaches (Created with Biorender.com (accessed on 13 February 2023)).

Cancer is the uncontrolled growth of disease in response to variations in the expression and state of various genes that give germinal and somatic cells a survival benefit and unconstrained proliferation capacity [18]. Alterations in three types of genes, namely tumor suppressor genes, DNA repair genes, and proto-oncogenes, contribute to the growth of cancer phenotypic features that restrain the innate death mechanism(s) integrated into cells, and also dysregulate cell proliferation occurrences. Cancer cells show a diverse range of genetic mutations, such as point mutations, gene rearrangements, and gene amplifications, which disrupt molecular signaling pathways that control survival, cell growth, and metastasis [19,20,21]. There is strong evidence that “epigenetic alterations”, such as DNA methylation and altered histone modification arrangements, which alter chromatin condensation and regulate the expression of a specific set of genes, cause cancer [22,23]. Every year, over 11 million people in the world are affected by cancer [24]. In 2022, the United States was expected to have 1,918,030 total incidences of cancer and 609,360 cancer-related fatalities. With around 350 mortalities each day, lung cancer is the leading cause of death in the US. Notwithstanding a 4% to 6% annual increment in cancer cases since 2011, the frequency of breast cancer persisted in rising slowly (by 0.5% annually) from 2014 to 2018, and the occurrence of prostate cancer remained steady [25] (Figure 1A).

Technologies that can identify and analyze the hallmarks of healthy cells and how they turn malignant have the potential to yield crucial information on the underlying pathology of cancer that might also contribute to early identification, diagnosis, and intervention. Biomarkers are critical for cancer diagnosis, prognosis, patient assessment, and treatment selection [26]. Technologies can be used to find the tumor’s location, including its subtype, stage, and therapeutic response. Recognition of such a pattern in neighboring cells, even in more distant and readily sampled regions of the body, can also influence cancer treatments. Biomarkers in clinical research could provide a deeper insight into the disease process. The search for biomarkers necessitates an in-depth understanding of the molecular mechanisms and cellular events that lead to cancer initiation, focusing on how small changes in several regulator proteins and genes may compromise several cellular functions. Finding the precise association between clinical pathology and cancer biomarkers while being able to identify tumors at an initial stage non-invasively is a critical concern [27].

Biomarkers are practical tools for detecting metastasis and recurrent malignant potential and tracking therapeutic outcomes in cancer patients undergoing cancer therapy and adjuvant therapeutics. Sulzyc-Bielicka et al. investigated thymidylate synthase gene polymorphism in colorectal cancer patients receiving adjuvant 5-fluorouracil. Individuals with overexpression of the thymidylate synthase gene had a considerably higher risk of early relapse of oral carcinoma in the post-intervention phase, as per their results [28]. Different types of cancer biomarkers are discussed in Table 1. The “discovery” strategy is commonly used to identify biomarkers. To rapidly discover single or sets of biomarkers, techniques including gene expression arrays, DNA arrays, polymerase chain reaction, and high-throughput sequencing are used (Figure 1B). A proper research approach, and thorough validation and testing, are essential aspects of biomarker discovery [29]. 

## 3. Biosensors: Diagnostic Devices to Detect Biomarkers

**Figure 2:**
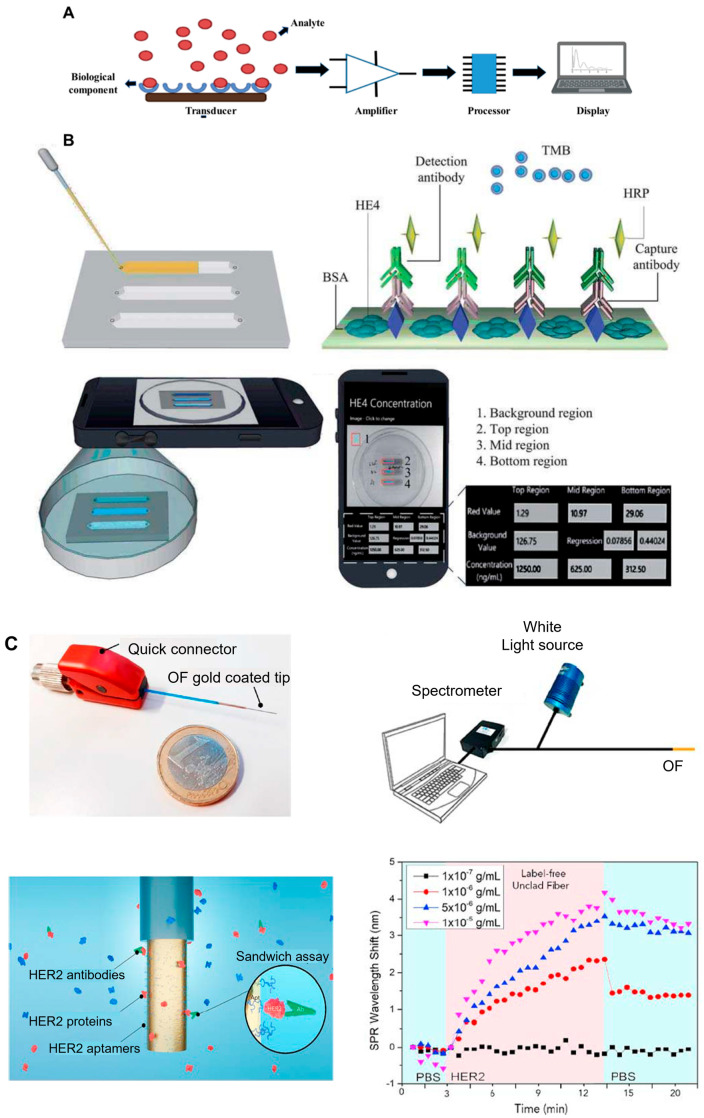

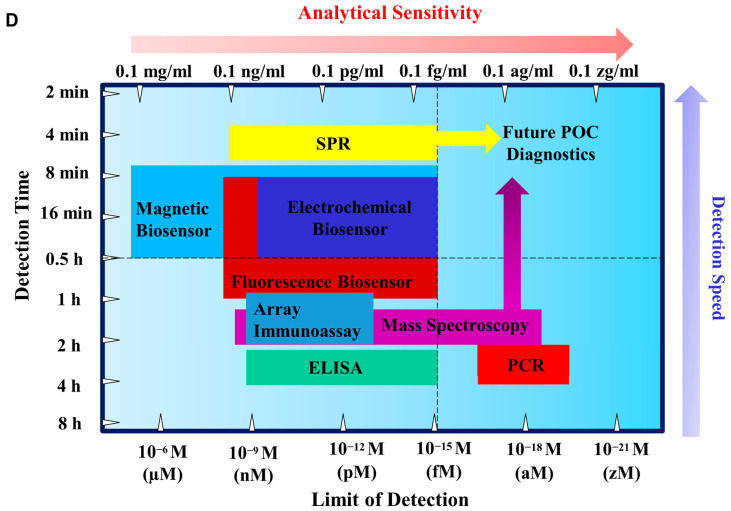
(**A**) Schematic representation of biosensors made of the following components: transducer, amplifier, processor, and display (created with Biorender.com). (**B**) A chip ELISA paired with a smartphone colorimetric diagnosis of ovarian cancer through urine is depicted in this diagram. A tiny quantity of urine was fed into the microchip on which sandwich ELISA was conducted. A cellphone’s installed camera was used to photograph the color generation in the chip. An integrated software device was used to measure the level of HE4 in urine. The pixel values from the designated region were recorded by the smartphone app. The level of the HE4 marker in each microchannel was estimated and shown on the device, relying on the regression of the calibration curves [62]. (**C**) Optical biosensor (surface plasmon resonance) was utilized to precisely identify HER2 proteins (in red) using a gold-coated fiber, with antibodies amplified in a sandwich arrangement (in green). To specifically target HER2, aptamers containing thiols are anchored on the surface. An optical fiber sensor is coupled to a spectrophotometer, and a laser source (480–720 nm) is shown in this schematic. The gadget is transportable and may be used with a laptop. A Gaussian surface plasmon resonance curve in PBS was generated using the gold-coated optical fiber [66]. (**D**) Schematic representation of comparison between the analytical sensitivity and detection time of different biosensing techniques.

A biosensor is an analytical device containing a molecular identification component linked to or combined with a transducer. Other components of biosensors are an amplifier and the signal processing unit (Figure 2A). Based on the different classes of transducers, the biosensor is classified as electrical, mass-based, electrochemical, or optical. Since electrochemical sensors are transportable, simple to use, inexpensive, and in most circumstances disposable, these are utilized in point-of-care devices (e.g., glucose sensors). Amperometry is the most common approach for detecting the current produced by an electrolyte ion in a biochemical reaction. The literature extensively uses this approach to identify potential cancer biomarkers and cancerous cells. The significant detection markers for lung cancer, MUC5AC and annexin II, were detected utilizing amperometric immunosensing techniques with a limit of detection (LOD) of 280 ± 8.0 pg/mL [59]. A graphene nanocomposite functionalized with gold nanoparticles (AuNPs) was produced to screen the human cervical cancer marker miR-21 [59,60]. A novel biosensor with a detection limit of 0.04–400 nM for detecting human phosphatase of regenerating liver-3, a prognosis marker for hepatocellular cancer, has been created [61]. One of the most extensively employed methods for screening cancer-specific protein markers is electrochemical enzyme-linked immunosorbent assay (ELISA). Wang et al. developed a low-cost microchip ELISA-based diagnostic unit, shown in Figure 2B, that utilizes a portable monitoring device to evaluate the ovarian cancer marker HE4 using urine. In urine specimens from patients with cancer, the HE4 concentration measured by a smartphone or lensless CCD device was higher than in control subjects (*p* < 0.001). The device, coupled with a smartphone app, has a specificity of 90% and a sensitivity of 89.5% [62].

Optical biosensors offer a non-invasive approach to detecting cancer biomarkers. Most cancer-derived substances, such as miRNA, CTCs, proteins, exosomes, and DNA, are employed in optical biosensors. Plasma, saliva, urine, serum, and blood can all be used to detect these components [63]. Colorimetric biosensors, surface plasmon resonance (SPR), fluorescence-based, and localized surface plasmon resonance (LSPR) are some optical sensing approaches. Colorimetric biosensors employ chemo-responsive dyes to assess absorbance and the color change that is apparent to the naked eye during the reaction with the sensing agent. The human platelet-derived growth factor-BB marker was detected using a pH-colorimetric biosensor incorporating glucose oxidase. Incorporating glucose oxidase promotes cancer biomarker recognition by disregarding crosstalk between various analysis processes and samples [64]. The detection of cancerous cells has been established by employing hybrid electrochemical and fluorescence-efficient wireless sensor polymeric dot–manganese oxide compounds (PD/MnO_2_). Their fluorescence intensity changes when polymeric dots react with the alkaline phosphatase in malignant cells [65]. Due to its durability, diversity, and adaptability, SPR-based detection has been gaining attention for cancer detection in recent times. An optical fiber biosensor has been used to diagnose breast cancer via the HER2 protein marker (Loyez et al.). A 50 nm Au film was conjugated on HER2 ssDNA aptamers for the detection as shown in Figure 2C [66]. Hahn et al. designed a tunable linker-based AuNP biosensor for prostate and breast cancer diagnosis by clumping NPs. The switchable linkers assist in amplifying the signals [67]. SPR biosensors have several benefits over traditional cancer detection techniques, including the ability to detect tumors rapidly in a label-free mode, in real time, in situ, and with higher sensitivity [68]. There are several other biosensors based on techniques such as mass spectroscopy and the magnetic sensing approach. The analytical sensitivity and detection time of various sensors are compared in Figure 2D. As shown in the figure, SPR sensors can achieve reasonable sensitivity and superior detection speed.

## 4. Surface Plasmon Resonance

**Figure 3:**
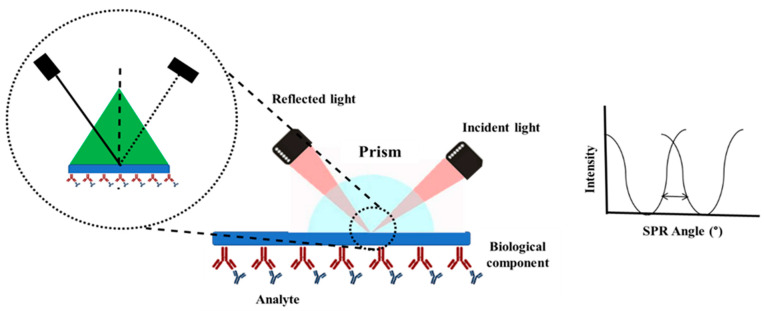
Schematic depiction of the SPR. A monochromatic laser is reflected on the surface. The plasmons generated by the surface are excited at a specific angle. The reflected light is continuously measured. This angle is affected by the analyte linked to the biological element on the surface. (Created with Biorender.com (accessed on 13 February 2023)).

Surface plasmons have been studied extensively since the 1960s. Kretschmann and Otto demonstrated the optical excitation of surface plasmons using the attenuated total reflection approach in the 1960s [69,70]. The phenomena of diffraction gratings induced by the stimulation of surface plasma waves (SPWs) were first defined by Wood [71]. When incident beam energy is linked to surface plasmons at the metal–dielectric junction, the SPR phenomenon occurs, leading the total internal reflection of the incident light to attenuate. An SPR sensor module typically consists of an optical device, a transducing medium that connects the biochemical and optical regions, and an electronic apparatus that supports the sensor’s optoelectronic elements and facilitates data processing. The SPR biosensor is classified as a refractometric device. The interaction of analytes with receptors on the sensor surface causes a local change in refractive index (Figure 3) and variation of the light propagation constant, resulting in a label-free real-time signal [72]. Silver or gold metal thin film on a glass slide can be used to detect biomarkers via the SPR effect.

SPR-based biosensors are frequently used with one of three SPR techniques: fluidic SPR, localized SPR (LSPR), or non-fluidic SPR imaging (SPRi). The interactions between photons and metallic nanoparticles are described as localized surface plasmon resonance (LSPR). Photons from incoming light interact with nanoparticles, yielding collective oscillation of non-propagating free electrons [73,74]. As the oscillation is locally limited to the surface of nanoparticles, any alteration in the localized dielectric milieu can impact the nanoparticle’s polarizability, driving the frequency of plasmon resonance to change and the optical extinction spectrum to shift [75,76]. The fluidic SPR is presently the most extensively used SPR technique in cancer diagnostics. In this form of SPR, the sensor part is placed in contact with a liquid solution, and measurements are taken. SPR sensors have been proven as a robust approach for determining molecular interactions, and the technology is expanding commercially. A variety of devices based on SPR are now being manufactured by several companies, such as Biacore AB, Jandratek GmbH, IBIS, BuoTul AG, and Affinity Sensors. Without the necessity for labeling, SPR-based technology enables real-time research of biomolecular interactions. SPR has made significant contributions to biosensors, sensing of numerous biomolecules, and real-time tracking of biological and chemical compounds. SPR’s capability to measure low molecular weight compounds makes it perfect for various application domains in pharmaceutical science, biosensing, environmental monitoring, and product safety [77]. SPR offers several advantages, including optimal speed, high efficiency, high precision, reproducibility, and real-time quantification [72]. Protein–nucleic acid, antibody–antigen, and ligand–receptor interactions are among the subjects of studies performed with these tools [78,79]. SPR instruments are employed to evaluate affinity constants (K_a_) and reaction kinetics (K_d_) for chemical reactions and the mechanism of ligand-to-receptor interaction.

### 4.1. Role of the Placement of Molecules on the Plasmonic Sensor

Broadly, energy transfer between donor and acceptor can be either a radiative or non-radiative process. Radiative energy transfer generally does not involve any interaction between donor and acceptor molecules to trigger the energy transfer (simple emission and absorption of a photon). On the other hand, non-radiative energy transfer such as Förster resonance energy transfer (FRET), Dexter energy transfer (DET), and plasmon resonance energy transfer (PRET) require a certain type of interaction mechanism to initiate the energy transfer. FRET is based on near field dipole–dipole coulombic interaction, DET involves electron exchange requiring overlap of the wavefunction of donor–acceptor molecules, and PRET is due to the dipole–dipole interaction between the plasmon dipole and the molecular dipole. The energy transfer processes are short ranged and the transfer efficiency decreases exponentially with distance. For example, if r is the donor–acceptor distance, DET has the shortest range of energy transfer with an exponential distance dependence, $e^{-2r/L}$, where L is the sum of the van der Waals radii of the donor and the acceptor. The energy transfer range of the DET process is ~1 nm. In FRET, the energy transfer process is proportional to $R_{0}^{6}/\left(R_{0}^{6}+r^{6}\right)$, where $R_{0}$ is the Förster distance. The energy transfer is limited by r ~10 nm. Similarly, PRET is proportional to $1/r^{n}$, where n is determined by the quantized dimensionality of the system. The energy transfer follows $r^{-6}$ dependence for a point dipole (e.g., quantum dots), $r^{-5}$ scaling for a 1D system such as line dipoles (e.g., nanowires), $r^{-4}$ dependence for 2D arrays of dipoles, and $r^{-3}$ dependence for point dipoles interacting with bulk dipoles (e.g., colloid nanoparticles) [80,81].

**Figure 4:**
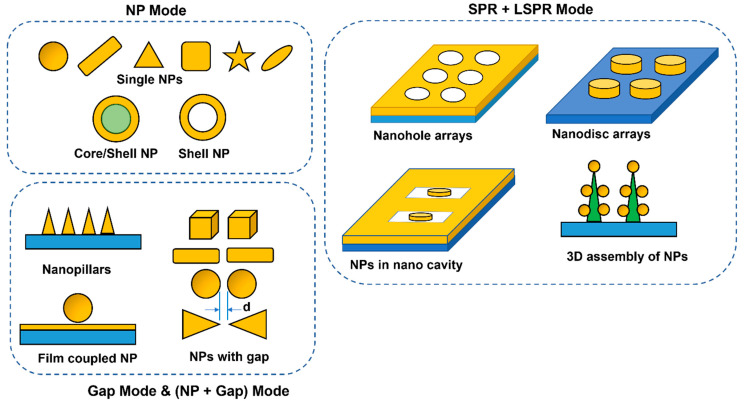
Schematic of nanoparticle (NP) images showing representative examples of localized surface plasmon resonance (LSPR or NP mode), gap mode, and combination of propagating surface plasmon resonance (SPR) and LSPR modes.

Figure 4 shows different plasmonic structures. The plasmon–molecule energy transfer process can be experimentally studied using a surface enhanced Raman scattering (SERS) process. During SERS, the excitation wavelength is enhanced due to the increase in the electric field near the surface of the nanoparticle (e.g., due to localized surface plasmon resonance, LSPR or NP mode). In addition, the emission wavelength is also enhanced during the SERS process due to availability of increased optical density of states for the molecule transition states. The increase in local electric field at the excitation wavelength can be represented by: $E_{loc}\left(\lambda_{ex}\right)=G_{1}E_{0}$, where $G_{1}$ is the enhancement factor at the excitation wavelength, and $E_{0}$ is the incident wavelength. The increase in local electric field at the emission wavelength can be represented by: $E_{loc}\left(\lambda_{em}\right)=G_{2}E_{0}$, where $G_{2}$ is the enhancement factor at the emission wavelength. The overall SERS intensity can be represented by: $I_{SERS}\propto {\left[E_{loc}\left(\lambda_{ex}\right)\right]}^{2}{\left[E_{loc}\left(\lambda_{em}\right)\right]}^{2}=G_{1}^{2}G_{2}^{2}$. If $G_{1}$ and $G_{2}$ are similar, we will expect an enhancement in intensity $\sim G^{4}$ where $G=E_{loc}/E_{0}$ = electric field enhancement at the plasmonic structure.

The local electric field enhancement is dependent on the position of the molecule from the surface of the NP (r), and the radius of the NP (a) [82]. The local electric field can be approximated by: $E_{loc}\left(r\right)\propto E_{0}{\left(1+\frac{r}{a}\right)}^{-3}$ [83,84,85]. Hence, the enhancement factor $G\sim {\left(1+r/a\right)}^{-3}$. Therefore, the SERS intensity will drop quickly with the increase in distance of the molecule from the NP surface with the relationship: $I_{SERS}\propto r^{-12}$. In order to improve the enhancement factor, one can implement the gap mode of the plasmonic NPs [86,87,88]. The local enhancement of electric field for two particles with diameters D and a gap between the two particles of d can be expressed as: $E_{loc}\propto E_{0}\left(D+d\right)/d$. Hence, the SERS enhancement factor will be: $I_{SERS}\propto \;{\left(1+D/d\right)}^{4}$. For example, for two NPs with diameter of 35 nm placed with a gap of 1 nm, the SERS enhancement factor will be ~$1.6\times 10^{6}$.

### 4.2. Role of Geometry of the Plasmonic Sensor

The scattering cross-section of a metal NP varies as follows:

$$\sigma_{abs}\left(\omega \right)=\frac{V}{3c}\varepsilon_{m}^{1.5}\overset{3}{\underset{i=1}{\sum }}\frac{1}{L_{i}^{2}}\frac{\omega \varepsilon_{im}}{{\left[\varepsilon_{r}+\varepsilon_{m}\left(\frac{1}{L_{i}}-1\right)\right]}^{2}+\varepsilon_{im}^{2}}$$

where V = volume of the NP = $4\pi a^{3}/3$, a = radius of the NP, c = speed of light in vacuum, $\varepsilon_{m}$ = dielectric function of the surrounding medium (of NP), ω = frequency of incident light, $L_{i}$ = shape factor (L = 1/3 for spherical particle, L = 1 for flat disc, L = 0 for infinite spheroid), $\varepsilon_{r}$ = real part of metal NP dielectric function, and $\varepsilon_{im}$ = imaginary part of metal NP dielectric function.

Since, $\sigma_{abs}\propto a^{3}$, the scattering will increase with an increase in the size of the particle. However, due to increased radiative emission, there will be increased radiative damping, and reduction of plasmon lifetime. This effect leads to an increase in plasmon linewidth or full-width-half-maximum (FWHM) of the plasmonic resonance peak. Usually, an increase in size (or diameter) of the NP leads to depolarization of the electromagnetic field across the NP, and the plasmon resonance wavelength shifts to a higher wavelength (red-shift of the resonance peak wavelength).

The shape effect of the NP on the plasmonic response can be understood with the following relationship:

$$\omega_{sp}=\sqrt{\frac{Ne^{2}}{\varepsilon_{0}m_{e}\left[\varepsilon_{r}+\left(\frac{1}{L}-1\right)\varepsilon_{m}\right]}},\;L=\frac{1-s^{2}}{s^{2}}\left(\frac{1}{2s}\ln\frac{1+s}{1-s}-1\right),\;\mathrm{and}\;s=\sqrt{1-{\left(\frac{1}{AR}\right)}^{2}}$$

where N = free electron density of metal, e = electron charge, $m_{e}$ = effective mass of electron, $\omega_{sp}$ = surface plasmon resonance frequency, AR = aspect ratio. When the aspect ratio (AR) increases, s will increase, and L will decrease. This will decrease $\omega_{sp}$, that is, there will be a red-shift in the plasmon resonance peak.

Potential drawbacks of SPR sensing include the ligand losing its native configuration after immobilization on the device surface, and its alignment preventing analyte interaction. Immobilization methods based on biotin, antibodies, and tags may aid in avoiding non-specific binding due to varying configurations of the entrapped ligand [89].

There are several pioneers in the field of SPR imaging, and they all have made tremendous contributions to the design and advancement of SPR. For instance, Corn et al. recently developed an SPRi detector for sequence-specific and rapid miRNA detection. SPR signals were strengthened using a method of consolidation of sequence-specific complexation of the miRNA to DNA, extension reaction of poly(A) polymerase by polyadenine (poly(A)) tails, complex formation of a ternary complex of T30–biotin/horseradish peroxidase–biotin/streptavidin to the poly(A) tails, and the oxidation responses of tetramethylbenzidine on the horseradish peroxidase by offering a blue precipitate on the surface, making it efficient and cost-effective [13].

Dr. Jiri Homola is another pioneer in the field of SPR imaging who has made immense advancements. He created a novel method for quickly activating the plasmonic properties of thin gold films punctured with nanohole arrays and combined with gold nanoparticle clusters for SPR detection of biomolecular binding [90]. Corso et al. consolidated angle-resolved SPR and SPRi in a single instrument, allowing optimization of the quantification prerequisites for SPRi [91]. Shao et al. created a prism-based 2D SPRi sensor with phase interrogation with a refractive index (RI) resolution of 2.7 × 10^−7^. To build a system for measuring phase retardation at a different wavelengths, they utilized a liquid crystal controllable filter to fluctuate the input spectrum and a liquid crystal phase modulation to initiate phase retardation among the s- and p-polarizations [92]. Botazzi et al. created a portable optical framework with an RI resolution of 4 × 10^−6^ by burying columns in a gold film [93]. Guner et al. demonstrated an SPRi system premised on a smartphone and an expendable grating coupler with an RI resolution of 4 × 10^−5^ [94]. Cappi et al. created an SPRi platform that uses gold nanoislands. To facilitate the spectral quantification, they used a white LED detector [95]. Zhang et al. created a small SPR hologram microscope that employed a Wollaston prism to incorporate the p-polarized light, which held the relevant data with linked s-polarized light; eventually, the results in an interferogram was utilized for SPRi reconstruction [96].

## 5. SPR in Cancer Detection

SPR has a substantial advantage over other optical detection approaches. It can detect both clear and colored specimens as the material’s turbidity does not change its sensing potential [97,98]. SPR-based sensors can effectively confirm the presence of targeted biomolecules in biological fluids such as blood, urine, saliva, or plasma at even low concentrations [99,100,101,102,103,104]. Early diagnosis and therapy are critical in the battle against cancers [105]. Highly sensitive and specific test outcomes can be acquired via next-generation sequencing (NGS), polymerase chain reaction (PCR), and enzyme-linked immunosorbent assay (ELISA) approaches for cancer diagnoses. However, the conventional techniques are tedious, involve multi-step sample preparation, and are time-consuming. SPR sensors with high sensitivity (picomolar level) are an alternative method to detect cancer biomarkers compared to conventional approaches [106,107].

We divided the results from the literature about the applications into two categories: SPR sensors validated against clinical samples and SPR sensors used to demonstrate preclinical proof-of-concept (non-validated) experiments. SPR approaches employing validated biosensing and associated analytical processes were used to detect certain cancer markers, indicated in a table at the end of the document. Springer and Homola devised an SPR biosensor-based detection method for carcinoembryonic antigen (CEA), a widely used indicator for the identification of colon cancer in blood serum [108]. The LOD was further improved from 8 ng/mL to diagnose colon cancers in clinical settings [108,109]. A sensor for determining carcinoma antigen 125 (CA 125) in serum samples was devised using the fluidic SPR approach. It leveraged an 11-mercaptoundecanoic acid coupling to attach anti-CA 125 antibodies to a gold surface using the EDS/NHS technique [110]. An enzyme-linked fluorescence test was utilized to validate the sensor by detecting CA 125 in a set of blood specimens simultaneously. The level of laminin 5 in the plasma of patients with bladder cancer is approximately three times greater than in healthy individuals. In parallel to the SPR experiments, laminin 5 detections were achieved by ELISA to validate the sensor. Wang et al. devised a sensor for detecting cytokeratin fragment 21-1 (CYFRA 21-1), a potential non-small cell lung carcinoma marker. The electrochemiluminescence approach was used to validate the sensor [111]. The non-validated SPR sensors are not currently being utilized for commercial purposes. In the next part of this review, we will discuss advances made over the years for screening of the various kinds of cancer markers through SPR and LSPR.

### 5.1. SPR for Detecting CTC and ctDNA

**Figure 5:**
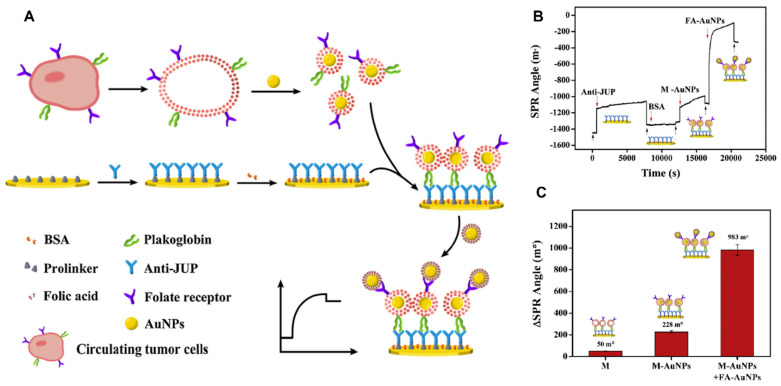
(**A**) A diagrammatic illustration of circulating tumor cells on the surface of the chip. (**B**) Sensorgram of the SPR, deionized water rinse procedure is represented by the black arrows, whilst the other modification procedures are represented by the red arrows. (**C**) Representation of SPR angle alteration when cancerous cell membrane alone, M-AuNPs, and FA-AuNPs–M-AuNPs are introduced to the anti-JUP engineered gold chip surface [116].

CTCs are well-known promising biomarkers that contribute to the detection and more precise profiling of many kinds of cancer by providing greater insight into the dynamic variations and features of the tumor [112]. A sensing approach relying on magnetic nanoparticle-based SPR has been reported for detecting folic acid and MUC-1 in breast cancer cells. In MCF-7 breast cancer cells, the folate receptor is overexpressed. This study employed human MUC-1 functionalized with cysteine aptamer-attached folic acid-linked magnetic nanoparticles to exclusively capture MCF-7 cells. The SPR angle increased as the quantity of MCF-7 cells increased, indicating that the MCF-7 cells were particularly trapped on the MUC-1 modified surface. The biosensor has a LOD of around 500 cells [113]. For label-free detection of living lung cancer cells, a three-dimensional multi-layered SPR biosensor relying on a DNA hybridization procedure was devised. The outer surface portion of the nanopillars (SU-8) in a three-dimensional biosensor, which comprised gold asymmetrical nanoholes and gold nanosquares incorporated in microfluidic systems, was potent in identifying living cancerous lung cells (A549) with LOD of 10^−7^ M while using a minimal clinical specimen volume (2 μL) [114]. The direct plasmon enhanced electrochemical (DPEE) approach has been developed for label-free ultra-sensitive measurement of CTCs in blood with high selectivity and LOD of 5 cells/mL by exploiting the impact of light intensity, LSPR wavelength, and temperature [115]. Gold nanostars (AuNSs) were bonded to a glassy carbon electrode that had been modified with an aptamer capable of capturing CTCs spiked in blood and serum samples. AuNSs boost the current responsiveness to electrocatalysis due to effective electron transmission via laser irradiation. To attain sensitive and specific detection of CTCs, Huang et al. devised the dual-selective method shown in Figure 5A. They synthesized and characterized folic acid-modified AuNPs (FA-AuNPs), and cell membrane fragment-modified AuNPs (M-AuNPs). Multi-signal amplifications, involving cell membrane fragments, M-AuNPs, and FA-AuNPs, were employed to detect CTCs via ultra-sensitive mode with LOD 10^1^–10^5^ cells/mL^−1^. CTC membranes express the specific junction plakoglobin (JUP), which is trapped on a gold chip customized using anti-JUP, which can be identified via a change in SPR angle, as shown in Figure 5B,C. Through the overexpression of FA receptors in the CTC surface, FA-AuNPs attach to M-AuNPs. This dual selectivity ensures the sensor’s reliability and sensitivity [116]. Tadimety et al. developed a gold nanorod-based nanoplasmonic technique for label-free screening of the mutated KRAS gene relevant to pancreatic cancer. Peptide nucleic acid (PNA) sequence-modified gold nanorods were exploited to detect G12V mutation associated with this KRAS gene. The LSPR peak was evaluated, demonstrating a LOD of 2 ng mL^−1^ [117].

### 5.2. SPR for Detection of miRNA

**Figure 6:**
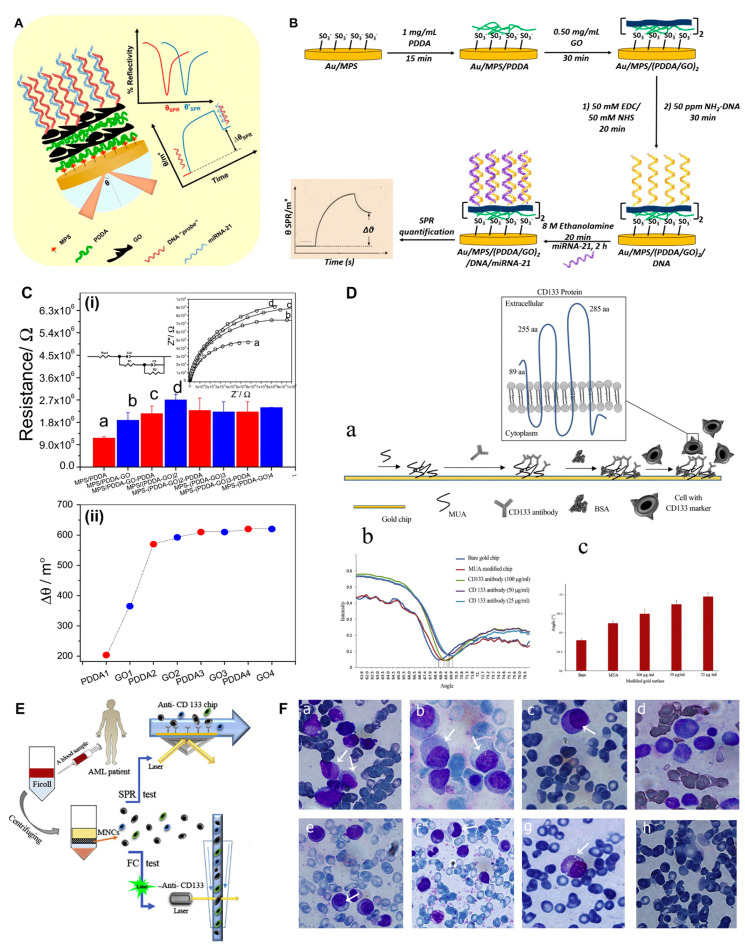
(**A**,**B**) Schematic depiction of several stages in the construction of the SPR miRNA-21 genosensor. Calculation of Δθ SPR from SPR sensogram acquired during developing miRNA-21 genosensor [124]. (**C**) i. For the Au/MPS system, bars show the fluctuation in overall Rct during the self-assembly of 1.00 mg mL^−1^ PDDA (red) and 0.50 mg/mL GO (blue). ii. SPR determined from sensorgram of 1.00 mg/mL PDDA (red) and 0.50 mg/mL GO (blue) sequentially. (**D**) **a.** A pictorial depiction of CD133 antibody attachment on a surface of gold; **b.** Sensorgrams acquired across several AML patients after injecting 1 × 10^5^ cells onto surface pre-incubated with anti-CD133; **c.** Change in the SPR angle with bare sensor surface, after MUA modification, and after incubation with different concentration of CD-133 antibodies. (**E**) Schematic showing the experimental set up. (**F**) The microscopic examination slides to demonstrate the presence of myeloblastic progenitor cells in AML subjects [125]. The white arrow indicates cytoplasm/nucleus region of the cell. Slides a, b, d, e, g are AML cells taken from bone marrow, and slides c, f are AML cells taken from peripheral blood. Slide h is cells from peripheral blood of normal patient and progenitor cells were not detected.

Cancer is linked to abnormal miRNA expression [118,119]. When discharged into circulation, miRNA is highly stable, rendering it an intriguing marker target. Zhang et al. (2017) devised ssDNA-modified Au nanocubes (AuNCs) for SPR-based identification with a LOD of 5 pM of miR-205, which is abundantly expressed in metastatic lung cancer [120]. An enzyme-aided target recycling process was formulated to detect gastric cancer-specific miRNA (miR-10b) in plasma and urine specimens with LOD 2.45 pM. The process comprised three steps: generating a DNA sandwich employing a sequence-specific hybridization reaction and Au nanotags encased with tannic acid-modified DNA enzyme-supported target recycling, and finally generating an enhanced LSPR reaction. miRNA-200 and miRNA-141 were detected in tumor cell extracts and serum specimens via an SPR-based sensor containing multiple layers of GO-Au NPs [121]. miRNA-141 was found at a low level of detection (LOD) of 0.1 fM, while miRNA-200 was detected with good selectivity using layers of GO-AuNPs and an accompanying dual amplification approach [122]. Xue et al. designed an SPR biosensor on a two-dimensional antimonide nanomaterial for the precise label-free identification of clinically significant markers in cancer, miRNA-155 and miRNA-21, with a detection limit of 10 aM, that is 2.3–10,000-fold greater than traditional miRNA detectors [123]. Mujica et al. devised an SPR-based nanosensor for detecting miRNA-21 (LOD—0.3 fM) from urine specimens, a cervical cancer prognostic biomarker. The sensing framework was generated by covalently binding a DNA probe onto two bilayers comprising poly(diallyldimethylammonium chloride (PDDA) and graphene oxide (GO) on a gold surface functionalized with 3-mercaptopropane sulfonate (MPS), as shown in Figure 6A. The field enhancer feature of GO was used to allow the probe DNA to be attached and to increase the sensitivity. Figure 6A shows the enhancement of Δθ in the SPR sensor after the hybridization of miRNA-21 [124].

### 5.3. SPR for Detecting Cancer Stem Cells

As described before, cancer stem cells are a potent biomarker in cancer detection. Fathi et al. developed a label-free, real-time SPR sensor for identifying cancer stem cells (CSCs) using the cell surface marker CD133, as illustrated in Figure 6B–F. The biosensor was utilized to identify this signal in certain individuals with acute myeloid leukemia (AML), and the findings were corroborated by flow cytometry data that revealed a strong connection. The current study examined the potential of SPR biosensors to capture CSCs. The researchers investigated isolated mononuclear cells from the bone marrow of AML patients based on CD133 expression. The rise in signal levels indicated in Figure 6B was produced by cancer stem cell capture on the altered surface [125].

### 5.4. SPR for Detection of Protein

**Figure 7:**
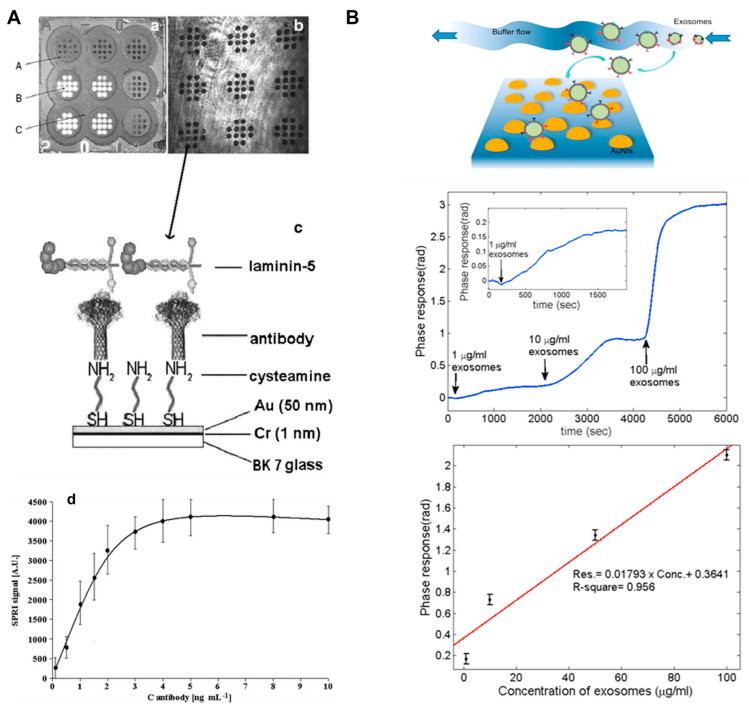

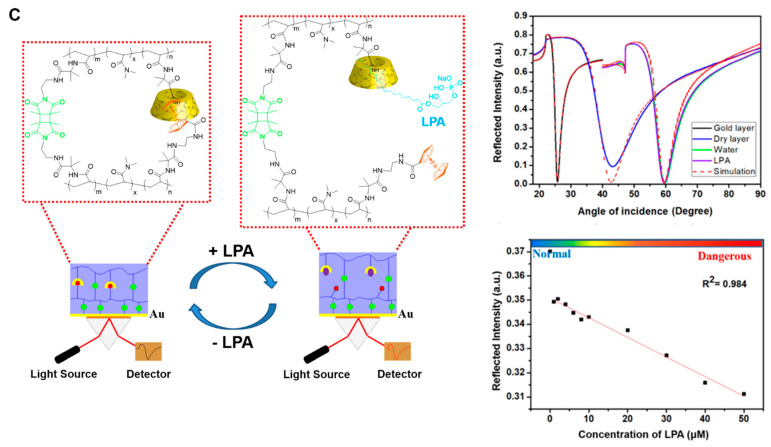
(**A**) (a) Image of the sensor (A—photopolymer; B—free gold surface; C—hydrophobic paint); (b) SPR photograph of the sensor acquired via a CCD camera. (c) The functional component of the device is depicted schematically [130]. (d) Change in the SPR angles with different concentration of antibodies. (**B**) A schematic depiction of biophysical interaction between exosome and SAM-AuNIs. Exosome detection sensitivity using LSPR. The study employed the LSPR sensitivity of three distinct exosomal concentrations (1 µg/mL, 10 µg/mL, and 100 µg/mL); the highest phase responsiveness was recorded at 100 µg/mL [140]. (**C**) The SPR-OWS sensor, which incorporates a dually crosslinked supramolecular hydrogel to identify LPA, is shown schematically [142].

Proteins are also potential markers for cancer detection, specifically circulating protein that leaves the tumor microenvironment and diffuses into the bloodstream. A microfluidic LSPR sensor device was used to identify four breast cancer protein markers (ErbB2, CA 125, CEA, and CA 15-3) in human blood [126]. The detection is much more sensitive than the standard enzyme-linked immunosorbent approach. Applying negative resistance electron beam lithography, a mercaptoundecanoic Au-SAM was built on a glass surface to construct the platform. Szymanska et al. developed an SPRi sensor to assess CA 125/MUC16 levels in ovarian cancer and endometrial cyst patients [127]. A cysteamine linker was utilized to covalently bind an Au chip to an anti-MUC16 antibody. The detection range of the CA 125/MUC16 sensor was 2.2–150 U/mL [127]. The cytokeratin 19 (CK19) marker was employed to identify lung cancer via an SPR sensor comprising graphene oxide modified with a carboxyl group (GO–COOH). The GO–COOH films were then mounted on gold chips using cystamine to produce sensor chips [128]. Finally, anti-CK19 antibodies were used to determine CK19 with a LOD of 0.05 pg/mL. Prostate-specific antigen (PSA), a prominent prostate cancer marker, was measured in serum using a combined colorimetric and SPR sensor with a LOD of 0.009 ng/mL [129]. To begin, triangular AuNPs were conjugated with a1 PSA-binding antibody. The complexes were then exposed to PSA molecules in the presence of two antibodies coated on magnetite nanoparticles for prostate cancer detection. Sankiewicz et al. devised the sensor shown in Figure 7A for the detection of laminin 5, an emergent cancer marker. The non-fluidic SPRi approach was applied with the sensor. The anti-laminin 5 antibodies were mounted on a series of gold measurement sites through a cysteamine coupler using the EDS/NHS method [130]. For detecting the HER2 marker in breast cancer, Loyez et al. designed an optical fiber-based SPR (OF-SPR) sensor. A sprayed gold layer was applied to optical fibers, leading to improved sensitivity to surface refractive index changes. In label-free mode, HER2 biomarkers were identified at 0.6 g/mL [66]. With the use of amino-modified Ti3C2-MXene (N-Ti3C2-MXene) nanosheets, Wu et al. established an ultra-sensitive SPR biosensor for detecting the cancer marker carcinoembryonic antigen (CEA) with LOD 1.7 pg mL^−1^. The nanosheets were modified utilizing (3-aminopropyl) triethoxysilane (APTES) to provide amino terminals for attaching anti-CEA antibody (Ab2) through the bond formation. The monoclonal anti-CEA antibody (Ab1) was fixed by employing a Staphylococcal protein A (SPA)-coated Au film as a sensor platform. After trapping CEA, the N-Ti3C2-MXene-Ab2 nanocomplex was added to the sensor system for producing sandwiched immunocomplexes upon this SPR chip [131].

### 5.5. SPR for Detection of Exosomes

Exosomes transport cargo, indicating genomic or signaling abnormalities in the tumor cells of origin [132,133,134]. In general, many exosomes in circulation are exploited to diagnose a particular form of cancer since they typically fit with SPR sensor depth, and label-free detection is often achievable [135,136,137]. For example, the identification of exosomes generated by MCF-10A (healthy breast cells) and MCF-7 (breast tumor cells) in fetal bovine serum was demonstrated utilizing an AuNP-based SPR aptasensor. Compared to the gold standard ELISA approach, an SPR-based detection showed a 104-fold reduction in LOD (5 × 10^3^ exosomes/mL). Furthermore, this SPR sensor was competent in differentiating between MCF-7 and MCF-10A [138]. Mao et al. devised a graphene customized gold chip-based SPR sensor that used a multi-functional peptide (M-Pep) as a recognition supermolecule (SS-IMVTESSDYSSY-KK-FHYQRDTPKSYN) to sense PD-L1 exosomes [139]. These PD-L1 exosomes are highly elevated by several cancer cells, including ovarian cancer, melanoma, and lung cancers. The fabricated M-Pep-based real-time SPR biosensor is sensitive to PD-L1 exosomes with a LOD of 20 particles/mL [139]. Figure 7B shows that multi-vesicular (MV) exosomes from A-549 cells were identified by LSPR in serum and urine specimens from a mouse model with lung cancer [140]. Self-assembled gold nano-islands on a glass surface formed the sensors. Figure 7B shows the LSPR sensitivity at distinct exosome concentrations, and the exosomes were quantified minimally at 0.194 µg/mL using this approach [140]. Sina et al. used a surface plasmon resonance (SPR) sensor with sensitivity as low as 8280 exosomes/L to show a simple method for label-free real-time diagnosis of BT474 (breast cancer cell) exosomes [141].

### 5.6. SPR for Detection of Lipids

As discussed previously, tumor cells show dysregulated lipid metabolism, and various lipid molecules have been exploited to detect various kinds of cancer. SPR is one of the potent techniques for screening of cancer-specific lipids. Li et al., for example, developed a dually crosslinked supramolecular hydrogel (DCSH) to trap lysophosphatidic acid (LPA), a biomarker for early-stage ovarian cancer. LPA, which serves as a guest molecule, binds with the host molecule β-cyclodextrin (β-CD) in a competitive manner, critical for the responsive action of the biosensor towards the LPA [142]. Ferrocene (Fc) also serves as a guest molecule that binds with β-CD. The target LPA, which serves as a competitive guest molecule, breaks the interaction between the actual host and guest pair (β-CD and Fc). SPR coupled with optical waveguide spectroscopy (SPR-OWS) was used to detect LPA with good selectivity and sensitivity (LOD—0.122 μM), suggesting DCSH as an SPR-OWS biosensor for detecting LPA in mimicked plasma (Figure 7C) [142].

## 6. Other Applications

Although SPR biosensors allow researchers to detect biomarkers in real-time label-free mode, innovative approaches are evolving to analyze thousands of interactions simultaneously. High-throughput SPR approaches, especially combined with innovative protein array techniques, offer tools for screening drug compounds, and developing antibodies for cancer therapy. In the next section, we discuss these two potent roles of SPR in cancer research.

### 6.1. SPR for High-Throughput Anti-Cancer Drug Screening

**Figure 8:**
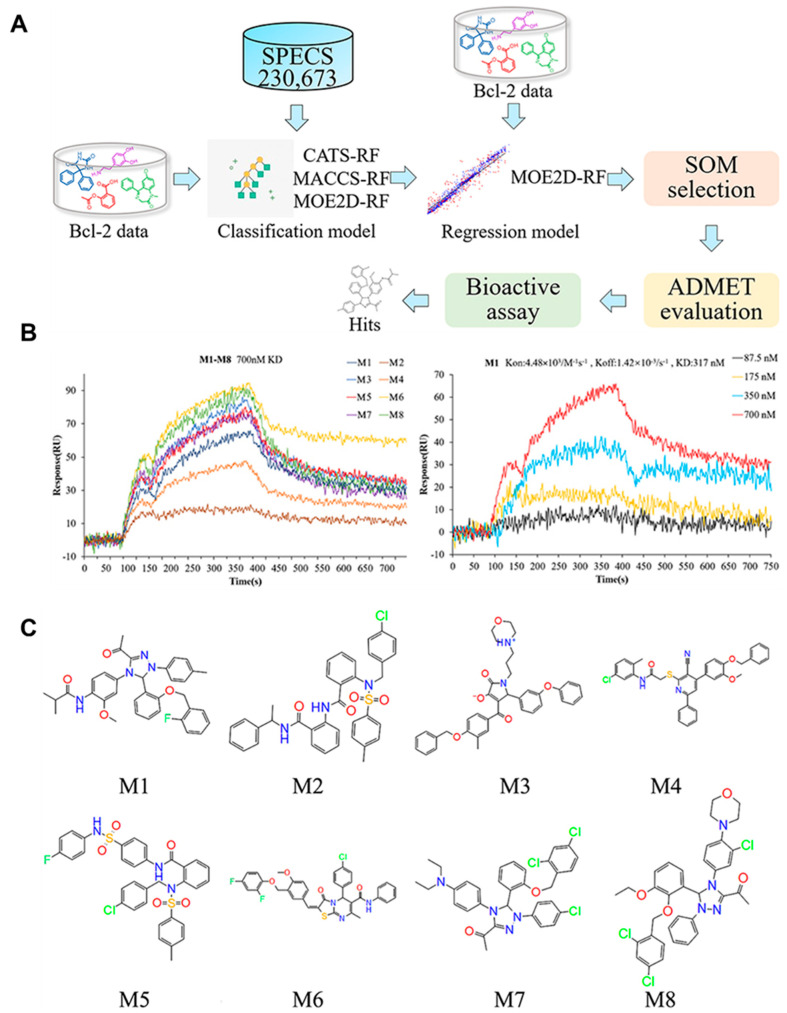
SPR technique was used to investigate the interaction between the potential drug candidates and Bcl-2. (**A**) Colored lines stand for the binding curves for the eight potential drug candidates. (**B**) Four colored lines indicate the binding curves for the various concentration gradients for M1. (**C**) Chemical compositions of the substances M1–M8 [148].

Initial phases in drug development entail target selection, testing, and optimization of target molecules. SPR is a promising tool for screening therapeutic candidates because it can detect interactions between minuscule compounds and fixed target proteins [143,144]. The adoption of SPR sensing devices (Biacore13000 and Biacore1S51) has three key benefits in the context of drug discovery. Tests are label-free, precisely monitoring and generating kinetic data on small molecules’ interaction with mounted therapeutic targets. For anti-cancer therapeutic screening, Loo et al. established an aptamer-based biobarcode (ABC) test to capture cytochrome-c (Cyto-c), a cell death indicator secreted by cancerous cells [145]. Micromagnetic particles (MMPs) functionalized with antibodies (Ab) and an aptamer selective to Cyto-c (MMP-Ab–Cyto-c–aptamer) were used to trap Cyto-c. The DNA biobarcode was hybridized with probes specifically engineered for RNase H for SPR detection. Phenylarsine oxide, which was screened by this ABC assay, appeared to be a potential therapeutic molecule to kill multi-drug-resistant liver cancer cells with a nanomolar concentration [145]. In a study, researchers used natural product enhanced DNA-encoded chemical libraries (nDELs) to test their anti-cancer effect. The target for nDEL was poly (ADP-ribose polymerase 1 (PARP1), and interaction screening was accomplished using SPR [146]. In BRCA-deficient cells, luteolin has the most potent antagonistic effect against PARP1 and triggers G2/M phase arrest and DNA double-strand breakage. All the findings indicate that inhibition of PARP1 is one of the pathways enabling luteolin’s anti-cancer effect [146]. Rizhen Huang et al. also developed naphthoquinone aromatic amide oxime compounds that can target both STAT3 and indoleamine-2,3-dioxygenase 1 (IDO1) for joint anti-cancer therapy and immunotherapy [147]. SPR confirmed that sample molecule 40 has a strong binding interaction for IDO1 and STAT3. In a mouse model, compound 40 has shown attenuated immunological response and tumor development, implying that it possesses joint immunomodulatory and anti-cancer actions [147]. Bcl-2 is a crucial regulator of apoptosis linked to cancer, making it a possible target for anti-cancer therapy. A research group performed a high-throughput screening strategy based on QSAR to find prospective Bcl-2 antagonists. An SPR binding experiment was used to explore the interaction between the Bcl-2 protein and the screened medications [148]. SPR binding experiments screened the anti-tumor actions of the eight substances (M1–M8), and the compound M1 was found to be a potential inhibitor for Bcl-2, shown in Figure 8A,B. M1 suppressed Bcl-2 expression and induced apoptosis in breast cancer cells through inducing mitochondrial malfunction, resulting in cytotoxicity [148].

### 6.2. SPR for Anti-Cancer Antibody Development

Therapeutic antibodies for cancer therapy are among the pharmaceutical industry’s fastest-growing segments; yet, their applicability has been limited due to immunogenicity issues. SPR has been a helpful technique for assessing therapeutic antibodies in recent times. Gassner et al. developed an SPR-based test to evaluate the binding ability of a bispecific-bivalent anti-Ang-2/anti-VEGF antibody that interacts with either vascular endothelial growth factor A (VEGF-A) or angiopoietin-2 (Ang-2), resulting in tumor growth suppression owing to diminished angiogenesis [149]. SPR was used to determine antibody response in the serum of 44 patients who had received injections of anti-A33, a colon cancer-targeting antibody. This finding suggests that SPR, as a method for generating therapeutic antibodies, might also be used to evaluate treatment efficacy [150]. 

A compilation of SPR approaches employing validated biosensing and associated analytical processes used to detect certain cancer markers are shown in Table 2. 

## 7. Conclusions

Clinical oncology is likely to embrace a revolutionary phase in which the molecular characteristics of the particular patient will increasingly dictate cancer detection, diagnosis, and therapy. The discovery and practical implementation of novel biomarkers will substantially impact cancer research. Biomarkers that can diagnose and predict cancer years before it appears symptomatic will be the game changer for cancer diagnosis and treatment. Such markers do not require tumor tissue for their detection, and they are secreted into the bloodstream by cancer cells, which will enable straightforward detection without even a minor surgical operation and will also be potential markers for population-based testing. SPR has been chosen over the conventional tools to detect cancer biomarkers due to several unique features, including real-time detection, being label-free, rapid monitoring, non-destructive examination, simple miniaturization, superior selectivity, cost-effectivity, reproducibility, and non-invasive diagnosis effects. This review highlighted some of the most relevant biomarkers in cancer diagnosis and the most advanced SPR and LSPR tools for detecting those tumor markers. The operational principles and applications of specific SPR, LSPR, and SPRi devices for the selective detection of various tumor markers were described. The assessed biosensors achieved low limit of detection (LOD) values for detecting cancer biomarkers in multiple sources, including serum, buffers, cell lines, and patient-derived samples. SPR-based screening approaches appear to be one of the most promising tools for high-throughput screening of anti-cancer drugs and therapeutic antibodies in the drug discovery sector because the interactions of the therapeutic molecule can be studied even at low concentrations.

SPR detection has been demonstrated to be efficacious in sensing major clinical molecules at the required sensitivity levels for diagnostic purposes. Even so, just 1% of SPR detecting publications have included the evaluation of clinical specimens. Early achievements of SPR sensors with clinical specimens should be assessed to offer a comprehensive vision of the field’s present status in order to expedite the transformation from proof-of-concept to real-time applications in clinical laboratories. The biomolecules detected in the majority of instances were at different concentrations of the nanomolar level, or above. The findings indicate significant advancements in the area of SPR detection for clinical diagnosis. There are several SPR instruments that are commercially available for detection including BIACORE, TI-SPR, SPR-670. The most impressive SPR feature is its ability to measure molecular binding kinetics with proteins (e.g., molecule–protein and protein–protein interactions). Although antibody and protein sensing has shown recent advances, there is a need to diagnose nucleic acids using SPR sensing in healthcare settings. SPR, which combines investigations in surface chemistry, chemical analysis, nanomaterials, systems engineering, and microfluidics, is poised to have a substantial effect on healthcare diagnostics in the coming years.

Considering the impressive relevance and effectiveness of SPR-based sensing devices for cancer marker diagnosis in recent years (Table 2), numerous challenges in the area of SPR sensing must be fixed before the SPR technique can be extensively exploited in clinical settings. SPR sensors must be designed to analyze potential markers in blood serum, plasma, and other body fluids to enhance their relevance in cancer detection and treatment. Future research should emphasize preparing a multi-purpose aptamer-based tool that can simultaneously detect and bind to cancer cells. Additional issues, including better sensitivity, reproducibility, and miniaturization, should be addressed to complete the SPR-based biosensing approach. SPR will be integrated with other techniques, emergent technologies, or innovative sensor materials, resulting in the emergence of long-term in vitro approaches and superior therapeutic candidates. An effective SPR approach for quickly evaluating a potential toxicological profile of a drug candidate could be created.

## Figures and Tables

**Table 1:** Example of potential biomarkers for different cancer types.

Biomarker	Biomarker Type	Sensitivity/Specificity and Predictive Value	Cancer Type	Source	Biological Concentration	Clinical Use	Conventional Technique	Sample No. (n)	Ref.
Alpha-fetoprotein (AFP)	Protein	Sensitivity: 65%/Specificity: 89%	Hepatocellular carcinomas	Serum	>400 ng/mL	Diagnostic and prognostic	Immunoassay	-----	[30]
Bladder tumor antigen (BTA)	Protein	Sensitivity: 83%/Specificity: 92%	Bladder cancer	Urine	-----	Monitoring	Immunoassay	220	[31]
BRCA-1 and BRCA-2 mutations	Genomic	Sensitivity: 80%/Specificity: 100%	Breast cancer	Blood	----	Prognosis	DNA sequencing	-----	[32]
Cancer antigen 19-9 (CA 19-9)	Protein	Sensitivity: 81%/Specificity: 90%	Pancreatic cancer	Serum	s100 U/mL	Diagnostic and prognostic	ELISA	1040	[33]
Cancer antigen 15-3 (CA 15-3)	Protein	Sensitivity: 31%/Specificity: 86%	Breast cancer	Serum	25 U/mL	Monitoring	Immunoassay	1342	[34]
Cancer antigen 125 (CA 125)	Glycoprotein	Sensitivity: 80%/Specificity: 99.6%	Ovarian cancer	Serum	35 units/mL	Detection, diagnosis, and prognosis	Immunoassay	-----	[35]
Carbohydrate antigen 27.29 (CA 27.29)	Protein	Sensitivity: 65%/Specificity: 100%	Breast cancer	Serum	>35 U/mL	Monitoring	Immunoassay	213	[36]
Carcinoembryonic antigen (CEA)	Protein	Sensitivity: 88.3%/Specificity: 46.2%	Lung cancer	Serum	8.2 ng/mL	Detection, diagnosis, and prognosis	Immunoassay	-----	[37]
CD133	Protein (cancer stem cell marker)	------	Acute myeloid leukemia	Cells	-------	Diagnostic, prognostic, and therapeutic	Flow cytometry	-------	
Cluster of differentiation 9 (CD9)	Exosomal protein	-----	Breast cancer	Cells	-----	Diagnostic	ELISA	-----	
Cluster of differentiation 147 (CD147)	Exosomal protein	-----	Colorectal cancer	Serum	103.59 pg/mL	Diagnostic	ELISA	108	
CD166	Protein	Sensitivity: 58.6%/Specificity: 78.9%	Pancreatic cancer	Serum	22 ng/mL	Prognosis	ELISA	600	[38]
Collagen IV	Protein	-----	Breast cancer	Serum	103 ng/mL	Diagnostic	ELISA	41	[39]
Cyclin-dependent kinase 4 (CDK4)	Protein	Sensitivity: 70%/Specificity: 69%	Lung, head, and neck cancers	Serum	>29.6 ng/µL	Diagnostic	Immunochemistry	-----	[40]
Cytokeratin 19	Protein	----	Non-small cell lung carcinoma	Serum	8.92 ± 9.95 mU/mL	Prognosis	ELISA (0.5 ng/mL)	-----	[41]
Cytokeratin 19 fragments (CYFRA 21-1)	Protein	-----	Non-small cell lung cancer	Serum	-----	Prognostic and predictive	ELISA	-----	
Cytochrome P450 mutations	Genomic	-----	Prostate and breast cancer	Blood	-----	Risk and assessment and prognosis	DNA sequencing	-----	
E-cadherin	Protein	Sensitivity: 74.3%/Specificity: 97.1%	Breast cancer	Serum	2218.9 ± 319.6 ng/mL	Diagnostic	ELISA	35	[42]
Estrogen receptor (ER)	Protein	Sensitivity: 99.7%/Specificity: 95.4%	Breast cancer	Tissue	-----	Prognosis and prediction	Immunohistochemistry	1569	[43]
Fibrin/fibrinogen degradation products (FDPs)	Protein	Sensitivity: 100%/Specificity: 80%	Bladder cancer	Serum	-----	Monitoring	Immunoassay	192	[44]
Glutathione S-transferase (GSTP1) polymorphisms	Genomic	-----	Breast and prostate cancer	Blood	-----	Risk assessment, prognosis, and treatment	PCR restriction fragment-length polymorphism assay (PCR-RFLP assay)	-----	
Glypican-1 (GPC-1)	Exosomal protein	Sensitivity: 76.92%/Specificity: 70.85%	Pancreatic cancer	Serum	8.75 ng/mL	Diagnosis	Mass spectrometry	595	[45]
Haptoglobin	Protein	Sensitivity: 63.9%/Specificity: 88.1%	Lung cancer	Serum	1.985 mg/mL	Diagnosis, therapy response	Immunoassay	205	[46]
HER2	Protein	Sensitivity: 98.7%/Specificity: 99.3%	Breast cancer	Serum	65.38 ± 37.92 ng/mL (serum)	Prognosis and treatment	FISH, PCR	1210	[47]
Human chorionic gonadotrophin (hCG)	Protein	-----	Ovarian and testicular cancer	Serum	1000–10,000 IU/L	Diagnostic	ELISA	------	[38,48]
Human epidermidis protein 4 (HE4)	Protein	Sensitivity: 76%/Specificity: 92%	Ovarian cancer	Serum	-----	Diagnostic	Chemiluminescent immunoassay	986	[49]
Interleukin 8 (IL-8)	Protein	----	Multiple cancers	Serum	-----	Diagnostic and prognostic	ELISA (1–3 pg mL^−1^)	-----	
Laminin 5	Protein	-----	Bladder cancer	Serum	-----	Diagnostic	ELISA	-----	
Lysophosphatidic acid (LPA)	Lipid	-----	Ovarian cancer	Serum	8.6 µ mol/L	Detection, diagnosis, and prognosis	Mass spectrometry	48	[50]
MCF-7 cells	Cells	------	Breast cancer	Tumor sample	-------	Diagnostic and prognostic	Immunocytometry	------	
Melanoma-associated antigen 3/6 (MAGE 3/6)	Protein	-----	Ovarian cancer	Plasma	-----	Prognostic and therapy monitoring	Western blots	-----	
miR-16, miR-181, miR-34a, and miR-125b	RNA	----	Malignant tumors	Serum	-----	Diagnosis	RT-qPCR	-----	
miR-205	RNA	Sensitivity: 78%/Specificity: 69%	Lung cancer	Serum	----	Diagnosis	RT-qPCR	-----	[36,51]
Nuclear matrix protein 22 (NMP-22)	Protein	Sensitivity: 83%/Specificity: 71%	Bladder cancer	Urine	10 U/mL	Screening and monitoring	Immunoassay	2951	[52]
Osteopontin	Genomic	-----	Ovarian Cancer	Blood	-----	Detection, diagnosis, and prognosis	Microarray	-----	
p53	Protein	Sensitivity: 81.1%/Specificity: 83.3%	Head and neck cancer	Serum	401 pg/mL	Prognosis	ELISA	------	[53]
Progesterone receptor (PR)	Protein	Sensitivity: 94.8%/Specificity: 92.6%;	Breast cancer	Tissue	-----	Prognosis and prediction	Immunohistochemistry	1347	[43]
Prostate-specific antigen (PSA)	Protein	Sensitivity: 82.1%/Specificity: 80.6%	Prostate	Serum	2.6–4.0 ng/mL	Diagnostic and prognostic	Immunoassay	136	[54]
Ras-related C3 botulinum (Rac1)	Protein	-----	Non-small cell lung cancer	Tumor Tissue	-----	Prognostic	Immunohistochemistry	-----	
Ras mutations	Genomic	-----	Colon and lung cancer	Blood	-----	Risk assessment	Short oligonucleotide mass analysis (SOMA)	-----	
Thyroglobulin (Tg)	Protein	------	Papillary and follicular thyroid cancer	Serum	>10 ng/mL	Diagnostic and prognostic	ELISA	72	[41,55]
Transforming growth factor β (TGFβ)	Protein	Sensitivity: 86.7%/Specificity: 100%	Malignant tumors	Serum	370 pg/mL	Diagnostic and prognostic	ELISA	180	[37,56]
Transforming growth factor beta 1 (TGFβ1)	Protein	-----	Ovarian cancer	Plasma	31.2–2000 pg/mL	Prognostic and therapy monitoring	Western blots	28	[57]
Vascular endothelial growth factor (VEGF)	Protein	Sensitivity: 50%/Specificity: >90%	Multiple cancers	Serum	92–390 pg/mL	Prognosis	ELISA	-----	[58]
v-kit Hardy-Zuckerman 4 feline sarcoma viral oncogene homolog (KIT)	Protein	-----	Gastrointestinal	Tissue	-----	Prediction, diagnosis, and selection of therapy	Immunohistochemistry	-----	

**Table 2:** Examples of potential SPR-based tools for detection of cancer biomarkers.

Biomarker	Type of Probe	Type of SPR	Clinical Sample	Linear Range	LOD	Reference
BRCA-1 and BRCA-2 Mutations	LSPR	Non-Validated	----	----	----	[151,152]
HER2	SPR with Optical Fiber	Non-Validated	----	10^−12^–10^−6^ g/mL	9.3 × 10^−9^/mL	[66]
	SPR Based on Extraordinary Optical Transmission (EOT)	Non-Validated	Human Serum	----	3.0 ng/mL	[153]
	SPR Direct Method	Non-Validated	Human Serum	----	3.8 ng/mL	[154]
	SPRi Direct Method	Non-Validated	Buffer	1–200 ng/mL	2.06 ng/mL	[155]
Carcinoembryonic Antigen (CEA)	SPR Fluidic	Validated	Serum	1–60 ng/mL	1.0 ng/mL	[156]
	LSPR	Non-Validated	Serum	1–1 × 10^6^ fM	94 fM	[157]
	SPR with Antibody	Non-Validated	Serum	25–800 ng/mL	6.2 ng/mL	[158]
	SPR Sandwich Assay	Non-Validated	Buffer	3–400 ng/mL	3 ng/mL	[159]
	SPR with Antibody	Non-Validated	Buffer Spiked Human Serum	25–800 ng/mL	6.2 ng/mL	[158]
	SPR with Direct Detection	Non-Validated	Buffer	8–1000 ng/mL	8 ng/mL	[108]
Prostate Specific Antigen (PSA)	LSPR	Non-Validated	Serum	----	0.71 pg/mL	[160]
	SPRi Signal Enhancement with Quantum Dots	Non-Validated	HBS Buffer	1 ng/mL–100 pg/mL	100 pg/mL	[161]
	LSPR Integrated with Microfluidics	Non-Validated	50% Human Serum	10–100 ng/mL	1 ng/mL	[162]
	SPR Enhancement due to Resonant Coupling between Au Thin film and AuNP	Non-Validated	----	0.1–100 ng/mL	0.1 ng/mL	[163]
	SPR Direct Detection and Enhancement using Sandwich Assay	Non-Validated	PSA Spiked In Human Serum	1 ng/mL–10 μg/mL	10 ng/mL	[164]
	SPRi Signal Enhancement using Pegylated CdSe/ZnS Quantum Dots	Non-Validated	PBS	100 μg/mL–10 fg/mL	10 fg/mL	[165]
Cancer Antigen 125 (CA 125)	Non-Fluidic SPRi	Validated	Serum	2.2–150 units/mL	0.66 units/mL	[127]
	SPR Capacitive System	Non-Validated	Human Serum	0.1–40 units/mL	0.1 units/mL	[110]
Alpha-Fetoprotein (AFP)	Fluidic SPR	Validated	Serum	----	0.1 ng/mL	[111]
	LSPR Integrated With Microfluidics	Non-Validated	50 % Human Serum	5–1000 ng/mL	500 pg/mL	[166]
	LSPR	Non-Validated	Serum	1 fM–1 × 10^6^ fM	91 fM	[157]
	SPR Signal Enhancement using Fe_3_O_4_@Au–antibody	Non-Validated	----	1.0–200.0 ng/mL	0.65 ng/mL	[66]
Mir-205	LSPR	Non-Validated	Serum	10 pM–1 μM	5 pM	[120]
mir-181, mir-125b, mir-34a, and mir-16	SPRi	Non-Validated	Erythrocyte Lysate	0.1–500 pM	0.5 pM	[167]
Human Chorionic Gonadotrophin (hCG)	SPR	Non-Validated	Blood	8.32–0.065 nM	0.065 nM	[27,168]
	SPR using Antibody–DNA Conjugated Array	Non-Validated	Buffer 10% Plasma	10–100 ng/mL	13 ng/mL	[102]
	SPR Signal Enhancement using Secondary Antibody	Non-Validated	Urine	46–415 miu/mL (milli-international units per milliliter)	46.4 miu/mL (milli-international units per milliliter)	[169]
	Combining SPRi with Polarization Contrast	Non-Validated	PBS	0.5–10 μg/mL	500 ng/mL	[170]
	SPR based on Single Strand/Oligo(Ethylene Glycol) Self-Assembled Monolayer	Non-Validated	----	1 μg/mL	----	[171]
Cancer Antigen 19-9 (CA 19-9)	Fluidic SPR	Non-Validated	----	400–192,000 units/mL	410.9 units/mL	[172]
Cancer Antigen 15-3 (CA 15-3)	SPR	Non-Validated	Pleural Fluid	----	0.025 units/mL	[173]
	SPR based on Au/ZnO Thin Film	Non-Validated	Saliva	40–300 units/mL	----	[174]
Cancer Antigen 125 (CA 125)	SPR and Capacitive System	Non-Validated	Human Serum	0.1–40 units/mL	0.1 units/mL	[110]
Thyroglobulin (Tg)	LSPR	Non-Validated	Serum	0.001–100,000 pg/mL	93.11 fg/mL	[175]
MCF-7 Cells	SPR	Non-Validated	Serum	10^4^–10^6^ cells/mL	500 cells/mL	[113]
Cd133	SPR	Non-Validated	Blood	----	1 × 10^5^ cells/mL	[125]
Glypican-1 (GPC-1)	LSPR	Non-Validated	Serum	10^3^ to 10^6^ particles/mL	400 particles/mL	[176,177]
Cluster of Differentiation 9 (CD9)	LSPRi	Non-Validated	Cells	----	----	[178]
Activated Leukocyte Cell Adhesion Molecule (ALCAM)	SPR based on Antibody-DNA Conjugate Array	Non-Validated	10% Blood Plasma	10–1000 ng/mL	45 ng/mL	[102]
	SPRi based on Antibody–Oligo(Ethylene Glycol) Array	Non-Validated	10% Human Serum	----	6 ng/mL	[102]
Haptoglobin	SPR	Non-Validated	Serum	----	----	[179,180,181,182,183]
Ras Mutations	SPRi	Non-Validated	Plasma	----	----	[184,185]
Lysophosphatidic Acid (LPA)	SPR	Non-Validated	Blood Plasma	2 to 30 μM	0.122 μM	[142,186]
Human Epidermidis Protein 4 (HE4)	Non-Fluidic LSPR	Validated	Serum	10–10,000 pM	4 pM	[187]
Laminin 5	Non-Fluidic SPRi	Validated	Serum	0.014–0.1 ng mL^−1^	4 pg mL^−1^	[130]
Cyclin-Dependent Kinase 4 (CDK4)	Fluidic SPR	Validated	Serum	----	----	[188]
Collagen IV	Non-Fluidic SPRi	Validated	Serum	10–300 ng/mM	2.4 ng mL^−1^	[189]
Ras-Related C3 Botulinum (Rac1)	Fluidic SPR	Validated	Serum	1 to 5 mmol/L	----	[190,191]
CYFRA 21-1	SPR Fluidic Chip	Validated	Serum	10^−1^ to 10^3^ ng/mL	0.1 ng/mL	[111]
Vascular Endothelial Growth Factor (VEGF)	LSPR	Non-Validated	-	Nanomolar Range	Nanomolar Range	[192]
	LSPR based on the Fluorophore-Conjugated Aptamer	Non-Validated	Diluted Serum and Saliva	1.25 pM–1.25 μM	----	[193]
Interleukin 8 (IL-8)	Fluidic SPR	Non-Validated	Saliva	0–2 nM	2.5 pM	[194,195]
Cytokeratin 19	Fluidic SPR	Non-Validated	Serum	1.6–128.3 ng/mL	0.05 pg/mL	[128]
E-Cadherin	Non-Fluidic SPR	Non-Validated	Serum	0–200 ng/mL	16 ng/mL	[196]
P53	LSPR	Non-Validated	Serum	----	----	[197]
Cd166	Fluidic SPR	Non-Validated	Serum	----	<1 ng/mL	[198]
Cytokeratin	LSPR	Non-Validated	----	----	14 pM	[199]
Antiasparaginase	SPR	Validated	Serum	----	500 pM	[200]
Immunoglobulins Kappa and Lambda	SPR	Non-Validated	Serum	----	----	[201]
Galectin-1	LSPR	Non-Validated	Serum	----	10^−13^ M	[202]
Lipocalin-2	SPR	Non-Validated	Serum	2.5–500 ng/mL	0.6 ng/mL	[203]
Podoplanin	SPRi	Non-Validated	Blood Plasma	0.25–1 ng/mL	15 ng/mL	[204]
p38αMAP kinase	SPR	Non-Validated	Serum	----	----	[205]
Cathepsin G	SPRi	Non-Validated	Blood	----	----	[206]
Epstein–Barr virus	SPR	Non-Validated	Serum	----	10 pg/mL	[207]

## Data Availability

Not applicable.

## References

[1] Aronson J.K., Ferner R.E. (2017). Biomarkers—A general review. Curr. Protoc. Pharmacol..

[2] Blackadar C.B. (2016). Historical review of the causes of cancer. World J. Clin. Oncol..

[3] Bray F., Ferlay J., Soerjomataram I., Siegel R.L., Torre L.A., Jemal A. (2018). Global cancer statistics 2018: GLOBOCAN estimates of incidence and mortality worldwide for 36 cancers in 185 countries. CA Cancer J. Clin..

[4] Mordente A., Meucci E., Martorana G.E., Silvestrini A. (2015). Cancer biomarkers discovery and validation: State of the art, problems and future perspectives. Adv. Exp. Med. Biol..

[5] Gorgannezhad L., Umer M., Islam M.N., Nguyen N.T., Shiddiky M.J.A. (2018). Circulating tumor DNA and liquid biopsy: Opportunities, challenges, and recent advances in detection technologies. Lab Chip.

[6] Khanmohammadi A., Aghaie A., Vahedi E., Qazvini A., Ghanei M., Afkhami A., Hajian A., Bagheri H. (2020). Electrochemical biosensors for the detection of lung cancer biomarkers: A review. Talanta.

[7] Xiao L., Zhu A., Xu Q., Chen Y., Xu J., Weng J. (2017). Colorimetric Biosensor for Detection of Cancer Biomarker by Au Nanoparticle-Decorated Bi2Se3 Nanosheets. ACS Appl. Mater. Interfaces.

[8] Chen Y., Yu Q., Duan X., Wu W., Zeng G. (2020). Phosphofructokinase-M inhibits cell growth via modulating the FOXO3 pathway in renal cell carcinoma cells. Biochem. Biophys. Res. Commun..

[9] Cai X., Zhang H., Yu X., Wang W. (2020). A microfluidic paper-based laser-induced fluorescence sensor based on duplex-specific nuclease amplification for selective and sensitive detection of miRNAs in cancer cells. Talanta.

[10] Qu J.H., Dillen A., Saeys W., Lammertyn J., Spasic D. (2020). Advancements in SPR biosensing technology: An overview of recent trends in smart layers design, multiplexing concepts, continuous monitoring and in vivo sensing. Anal. Chim. Acta.

[11] Mahmoudpour M., Ezzati Nazhad Dolatabadi J., Torbati M., Pirpour Tazehkand A., Homayouni-Rad A., de la Guardia M. (2019). Nanomaterials and new biorecognition molecules based surface plasmon resonance biosensors for mycotoxin detection. Biosens. Bioelectron..

[12] Masson J.F. (2017). Surface Plasmon Resonance Clinical Biosensors for Medical Diagnostics. ACS Sens..

[13] Jebelli A., Oroojalian F., Fathi F., Mokhtarzadeh A., de la Guardia M. (2020). Recent advances in surface plasmon resonance biosensors for microRNAs detection. Biosens. Bioelectron..

[14] Homola J., Yee S.S., Gauglitz G. (1999). Surface plasmon resonance sensors: Review. Sens. Actuators B Chem..

[15] He L., Pagneux Q., Larroulet I., Serrano A.Y., Pesquera A., Zurutuza A., Mandler D., Boukherroub R., Szunerits S. (2017). Label-free femtomolar cancer biomarker detection in human serum using graphene-coated surface plasmon resonance chips. Biosens. Bioelectron..

[16] Patil P.O., Pandey G.R., Patil A.G., Borse V.B., Deshmukh P.K., Patil D.R., Tade R.S., Nangare S.N., Khan Z.G., Patil A.M. (2019). Graphene-based nanocomposites for sensitivity enhancement of surface plasmon resonance sensor for biological and chemical sensing: A review. Biosens. Bioelectron..

[17] Shao B., Xiao Z. (2020). Recent achievements in exosomal biomarkers detection by nanomaterials-based optical biosensors—A review. Anal. Chim. Acta.

[18] Mesri E.A., Feitelson M.A., Munger K. (2014). Human viral oncogenesis: A cancer hallmarks analysis. Cell Host Microbe.

[19] Weissleder R., Ntziachristos V. (2003). Shedding light onto live molecular targets. Nat. Med..

[20] Sidransky D. (2002). Emerging molecular markers of cancer. Nat. Rev. Cancer.

[21] Vogelstein B., Kinzler K.W. (2004). Cancer genes and the pathways they control. Nat. Med..

[22] Baylin S.B., Ohm J.E. (2006). Epigenetic gene silencing in cancer—A mechanism for early oncogenic pathway addiction?. Nat. Rev. Cancer.

[23] Jaenisch R., Bird A. (2003). Epigenetic regulation of gene expression: How the genome integrates intrinsic and environmental signals. Nat. Genet..

[24] Cho W.C.S. (2007). Contribution of oncoproteomics to cancer biomarker discovery. Mol. Cancer.

[25] Siegel R.L., Miller K.D., Fuchs H.E., Jemal A. (2022). Cancer statistics. CA Cancer J. Clin..

[26] Ludwig J.A., Weinstein J.N. (2005). Biomarkers in cancer staging, prognosis and treatment selection. Nat. Rev. Cancer.

[27] Bhatt A.N., Mathur R., Farooque A., Verma A., Dwarakanath B.S. (2010). Cancer biomarkers—Current perspectives. Indian J. Med. Res..

[28] Sulzyc-Bielicka V., Bielicki D., Binczak-Kuleta A., Kaczmarczyk M., Pioch W., Machoy-Mokrzynska A., Ciechanowicz A., Gołȩbiewska M., Drozdzik M. (2013). Thymidylate synthase gene polymorphism and survival of colorectal cancer patients receiving adjuvant 5-fluorouracil. Genet. Test. Mol. Biomarkers.

[29] Henry N.L., Hayes D.F. (2012). Cancer biomarkers. Mol. Oncol..

[30] Zhou L., Liu J., Luo F. (2006). Serum tumor markers for detection of hepatocellular carcinoma. World J. Gastroenterol..

[31] Sarosdy F., Hudson M.L.A., Ellis W.J., Soloway M.S., White R.D., Sheinfeld J., Jarowenko M.V., Schellhammer P.F., Schervish E.D.W., Patel J.A.Y.V. (1995). Using the Bard Bta Stat Test Cancer. Urology.

[32] Vaz F.H., Machado P.M., Brandão R.D., Laranjeira C.T., Eugénio J.S., Fernandes A.H., André S.P. (2007). Familial breast/ovarian cancer and BRCA1/2 genetic screening: The role of immunohistochemistry as an additional method in the selection of patients. J. Histochem. Cytochem..

[33] Ballehaninna U.K., Chamberlain R.S. (2011). Serum CA 19-9 as a Biomarker for Pancreatic Cancer-A Comprehensive Review. Indian J. Surg. Oncol..

[34] Safi F., Kohler I., Rottinger E., Suhr P., Beger H.G. (1989). Comparison of CA 15-3 and CEA in diagnosis and monitoring of breast cancer. Int. J. Biol. Markers.

[35] Scholler N., MUrban N. (2007). CA125 in ovarian cancer. Biomark. Med..

[36] Frenette P.S., Thirlwell M.P., Trudeau M., Thomson D.M.P., Joseph L., Shuster J.S. (1994). The diagnostic value of CA 27-29, CA 15-3, mucin-like carcinoma antigen, carcinoembryonic antigen and CA 19-9 in breast and gastrointestinal malignancies. Tumor Biol..

[37] Uygur M.M., Gümüş M. (2021). The utility of serum tumor markers CEA and CA 15–3 for breast cancer prognosis and their association with clinicopathological parameters. Cancer Treat. Res. Commun..

[38] Tachezy M., Zander H., Marx A.H., Stahl P.R., Gebauer F., Izbicki J.R., Bockhorn M. (2012). ALCAM (CD166) expression and serum levels in pancreatic cancer. PLoS ONE.

[39] Lindgren M., Jansson M., Tavelin B., Dirix L., Vermeulen P., Nyström H. (2021). Type IV collagen as a potential biomarker of metastatic breast cancer. Clin. Exp. Metastasis.

[40] Banerjee J., Pradhan R., Gupta A., Kumar R., Sahu V., Upadhyay A.D., Chaterjee P., Dwivedi S., Dey S., Dey A.B. (2016). CDK4 in lung, and head and neck cancers in old age: Evaluation as a biomarker. Clin. Transl. Oncol..

[41] Gao J., Lv F., Li J., Wu Z., Qi J. (2014). Serum cytokeratin 19 fragment, CK19-2G2, as a newly identified biomarker for lung cancer. PLoS ONE.

[42] Liang Z., Sun X.Y., Xu L.C., Fu R.Z. (2014). Abnormal expression of serum soluble e-cadherin is correlated with clinicopathological features and prognosis of breast cancer. Med. Sci. Monit..

[43] Dekker T.J.A., ter Borg S., Hooijer G.K.J., Meijer S.L., Wesseling J., Boers J.E., Schuuring E., Bart J., van Gorp J., Bult P. (2015). Quality assessment of estrogen receptor and progesterone receptor testing in breast cancer using a tissue microarray-based approach. Breast Cancer Res. Treat..

[44] Schmetter B.S., Habicht K.K., Lamm D.L., Morales A., Bander N.H., Grossman H.B., Hanna M.G., Silberman S.R., Butman B.T. (1997). A multicenter trial evaluation of the fibrin/fibrinogen degradation products test for detection and monitoring of bladder cancer. J. Urol..

[45] Zhou C., Dong Y., Sun X., Sui X., Zhu H., Zhao Y., Zhang Y., Mason C., Zhu Q., Han S. (2018). High levels of serum glypican-1 indicate poor prognosis in pancreatic ductal adenocarcinoma. Cancer Med..

[46] Lu J., Wang Y., Yan M., Feng P., Yuan L., Cai Y., Xia X., Liu M., Luo J., Li L. (2016). High serum haptoglobin level is associated with tumor progression and predicts poor prognosis in non-small cell lung cancer. Oncotarget.

[47] Dekker T.J.A., Borg S.T., Hooijer G.K.J., Meijer S.L., Wesseling J., Boers J.E., Schuuring E., Bart J., van Gorp J., Mesker W.E. (2012). Determining sensitivity and specificity of HER2 testing in breast cancer using a tissue micro-array approach. Breast Cancer Res..

[48] Lenhard M., Tsvilina A., Schumacher L., Kupka M., Ditsch N., Mayr D., Friese K., Jeschke U. (2012). Human chorionic gonadotropin and its relation to grade, stage and patient survival in ovarian cancer. BMC Cancer.

[49] Moore R.G., Brown A.K., Miller M.C., Badgwell D., Lu Z., Allard W.J., Granai C.O., Bast R.C., Lu K. (2008). Utility of a novel serum tumor biomarker HE4 in patients with endometrioid adenocarcinoma of the uterus. Gynecol. Oncol..

[50] Xu Y., Shen Z., Wiper D.W., Wu M., Morton R.E., Elson P., Kennedy A.W., Belinson J., Markman M., Casey G. (1998). Lysophosphatidic acid as a potential biomarker for ovarian and other gynecologic cancers. J. Am. Med. Assoc..

[51] Li L., Wang H., Lu Y., Pan Z. (2015). The expression and clinical significance of serum miR-205 for breast cancer and its role in detection of human cancers. Int. J. Clin. Exp. Med..

[52] Shariat S.F., Marberger M.J., Lotan Y., Sanchez-Carbayo M., Zippe C., Lüdecke G., Boman H., Sawczuk I., Friedrich M.G., Casella R. (2006). Variability in the Performance of Nuclear Matrix Protein 22 for the Detection of Bladder Cancer. J. Urol..

[53] Khan A.S., Ahmad S., Ullah Z., Sadiq N., Haq M., Sheikh A.K. (2022). Predictive value of tissue p53 protein expression and serum p53 antibodies in oral potentially malignant disorders: Relative to oral squamous cell carcinoma. J. Taibah Univ. Med. Sci..

[54] Al Saidi S.S., Al Riyami N.B., Al Marhoon M.S., Al Saraf M.S., Al Busaidi S.S., Bayoumi R., Mula-Abed W.A.S. (2017). Validity of prostate health index and percentage of [-2] pro-prostate-specific antigen as novel biomarkers in the diagnosis of prostate cancer: Omani tertiary hospitals experience. Oman Med. J..

[55] Der Lin J., Huang M.J., Hsu B.R.S., Chao T.C., Hsueh C., Liu F.H., Liou M.J., Weng H.F. (2002). Significance of postoperative serum thyroglobulin levels in patients with papillary and follicular thyroid carcinomas. J. Surg. Oncol..

[56] Shehata F., Monem N.A., Sakr M., Kasem S., Balbaa M. (2013). Epidermal growth factor, its receptor and transforming growth factor-β1 in the diagnosis of HCV-induced hepatocellular carcinoma. Med. Oncol..

[57] Santin A.D., Bellone S., Ravaggi A., Roman J., Smith C.V., Pecorelli S., Cannon M.J., Parham G.P. (2001). Increased levels of interleukin-10 and transforming growth factor-β in the plasma and ascitic fluid of patients with advanced ovarian cancer. Br. J. Obstet. Gynaecol..

[58] Kut C., Mac Gabhann F., Popel A.S. (2007). Where is VEGF in the body? A meta-analysis of VEGF distribution in cancer. Br. J. Cancer.

[59] Kim D.M., Noh H.B., Park D.S., Ryu S.H., Koo J.S., Shim Y.B. (2009). Immunosensors for detection of Annexin II and MUC5AC for early diagnosis of lung cancer. Biosens. Bioelectron..

[60] Grieshaber D., MacKenzie R., Vörös J., Reimhult E. (2008). Electrochemical biosensors—Sensor principles and architectures. Sensors.

[61] Jia Y., Qin M., Zhang H., Niu W., Li X., Wang L., Li X., Bai Y., Cao Y., Feng X. (2007). Label-free biosensor: A novel phage-modified Light Addressable Potentiometric Sensor system for cancer cell monitoring. Biosens. Bioelectron..

[62] Wang S., Zhao X., Khimji I., Akbas R., Qiu W., Edwards D., Cramer D.W., Ye B., Demirci U. (2011). Integration of cell phone imaging with microchip ELISA to detect ovarian cancer HE4 biomarker in urine at the point-of-care. Lab Chip.

[63] Underwood J.J., Quadri R.S., Kalva S.P., Shah H., Sanjeeviah A.R., Beg M.S., Sutphin P.D. (2020). Liquid biopsy for cancer: Review and implications for the radiologist. Radiology.

[64] Miao X., Zhu Z., Jia H., Lu C., Liu X., Mao D., Chen G. (2020). Colorimetric detection of cancer biomarker based on enzyme enrichment and pH sensing. Sens. Actuators B Chem..

[65] Won H.J., Robby A.I., Jhon H.S., In I., Ryu J.H., Park S.Y. (2020). Wireless label-free electrochemical detection of cancer cells by MnO_2_-Decorated polymer dots. Sens. Actuators B Chem..

[66] Loyez M., Lobry M., Hassan E.M., DeRosa M.C., Caucheteur C., Wattiez R. (2021). HER2 breast cancer biomarker detection using a sandwich optical fiber assay. Talanta.

[67] Hahn J., Kim E., You Y., Choi Y.J. (2019). Colorimetric switchable linker-based bioassay for ultrasensitive detection of prostate-specific antigen as a cancer biomarker. Analyst.

[68] Prasad A., Chaichi A., Mahigir A., Sahu S.P., Ganta D., Veronis G., Gartia M.R. (2020). Ripple mediated surface enhanced Raman spectroscopy on graphene. Carbon..

[69] Kretschmann E., Raether H. (1968). Radiative Decay of Non Radiative Surface Plasmons Excited by Light. Z. Fur Naturforsch.-Sect. A J. Phys. Sci..

[70] Otto A. (1968). Excitation of nonradiative surface plasma waves in silver by the method of frustrated total reflection. Z. Für Phys..

[71] Wood R.W. (1901). On a remarkable case of uneven distribution of light in a diffraction grating spectrum. Proc. Phys. Soc. Lond..

[72] Homola J. (2003). Present and future of surface plasmon resonance biosensors. Anal. Bioanal. Chem..

[73] Willets K.A., Van Duyne R.P. (2007). Localized surface plasmon resonance spectroscopy and sensing. Annu. Rev. Phys. Chem..

[74] Ferhan A.R., Jackman J.A., Cho N.J. (2016). Integration of quartz crystal microbalance-dissipation and reflection-mode localized surface plasmon resonance sensors for biomacromolecular interaction analysis. Anal. Chem..

[75] Stewart M.E., Anderton C.R., Thompson L.B., Maria J., Gray S.K., Rogers J.A., Nuzzo R.G. (2008). Nanostructured plasmonic sensors. Chem. Rev..

[76] Yang J., Giessen H., Lalanne P. (2015). Simple analytical expression for the peak-frequency shifts of plasmonic resonances for sensing. Nano Lett..

[77] Rezabakhsh A., Rahbarghazi R., Fathi F. (2020). Surface plasmon resonance biosensors for detection of Alzheimer’s biomarkers; an effective step in early and accurate diagnosis. Biosens. Bioelectron..

[78] Achen M.G., Roufail S., Domagala T., Catimel B., Nice E.C., Geleick D.M., Murphy R., Scott A.M., Caesar C., Makinen T. (2000). Monoclonal antibodies to vascular endothelial growth factor-D block its interactions with both VEGF receptor-2 and VEGF receptor-3. Eur. J. Biochem..

[79] Vogel M., Miescher S., Kuhn S., Zürcher A.W., Stadler M.B., Ruf C., Effenberger F., Kricek F., Stadler B.M. (2000). Mimicry of human IgE epitopes by anti-idiotypic antibodies. J. Mol. Biol..

[80] Namgung S., Mohr D.A., Yoo D., Bharadwaj P., Koester S.J., Oh S.H. (2018). Ultrasmall Plasmonic Single Nanoparticle Light Source Driven by a Graphene Tunnel Junction. ACS Nano.

[81] Lee Y., Kamal A.S.A., Abasaki M., Ho Y.L., Takakura Y., Delaunay J.J. (2016). Gap Plasmons Multiple Mirroring from Spheres in Pyramids for Surface-Enhanced Raman Scattering. ACS Photonics.

[82] Chen H., Shao L., Li Q., Wang J. (2013). Gold nanorods and their plasmonic properties. Chem. Soc. Rev..

[83] Ding S.Y., Yi J., Li J.F., Ren B., Wu D.Y., Panneerselvam R., Tian Z.Q. (2016). Nanostructure-based plasmon-enhanced Raman spectroscopy for surface analysis of materials. Nat. Rev. Mater..

[84] Kennedy B.J., Spaeth S., Dickey M., Carron K.T. (1999). Substrates Based on Self-Assembled Monolayers Formed Using Alkanethiols. J. Phys. Chem. B.

[85] Masango S.S., Hackler R.A., Large N., Henry A.I., McAnally M.O., Schatz G.C., Stair P.C., Van Duyne R.P. (2016). High-Resolution Distance Dependence Study of Surface-Enhanced Raman Scattering Enabled by Atomic Layer Deposition. Nano Lett..

[86] Devaraj V., Lee J.M., Kim Y.J., Jeong H., Oh J.W. (2021). Engineering efficient self-assembled plasmonic nanostructures by configuring metallic nanoparticle’s morphology. Int. J. Mol. Sci..

[87] Zong C., Xu M., Xu L.J., Wei T., Ma X., Zheng X.S., Hu R., Ren B. (2018). Surface-Enhanced Raman Spectroscopy for Bioanalysis: Reliability and Challenges. Chem. Rev..

[88] Devaraj V., Lee J.M., Oh J.W. (2018). Distinguishable plasmonic nanoparticle and gap mode properties in a silver nanoparticle on a gold film system using three-dimensional FDTD simulations. Nanomaterials.

[89] Helmerhorst E., Chandler D.J., Nussio M., Mamotte C.D. (2012). Real-time and label-free bio-sensing of molecular interactions by surface plasmon resonance: A laboratory medicine perspective. Clin. Biochem. Rev..

[90] Kotlarek D., Fossati S., Venugopalan P., Gisbert Quilis N., Slabý J., Homola J., Lequeux M., Amiard F., Lamy De La Chapelle M., Jonas U. (2020). Actuated plasmonic nanohole arrays for sensing and optical spectroscopy applications. Nanoscale.

[91] Corso A.J., Zuccon S., Zuppella P., Pelizzo M.G. (2015). Flexible SPR system able to switch between Kretschmann and SPRi. Opt. Sens..

[92] Shao Y., Li Y., Gu D., Zhang K., Qu J., He J., Li X., Wu S.-Y., Ho H.-P., Somekh M.G. (2013). Wavelength-multiplexing phase-sensitive surface plasmon imaging sensor. Opt. Lett..

[93] Bottazzi B., Fornasari L., Frangolho A., Giudicatti S., Mantovani A., Marabelli F., Marchesini G., Pellacani P., Therisod R., Valsesia A. (2014). Multiplexed label-free optical biosensor for medical diagnostics. J. Biomed. Opt..

[94] Guner H., Ozgur E., Kokturk G., Celik M., Esen E., Topal A.E., Ayas S., Uludag Y., Elbuken C., Dana A. (2017). A smartphone based surface plasmon resonance imaging (SPRi) platform for on-site biodetection. Sens. Actuators B Chem..

[95] Cappi G., Spiga F.M., Moncada Y., Ferretti A., Beyeler M., Bianchessi M., Decosterd L., Buclin T., Guiducci C. (2015). Label-Free detection of tobramycin in serum by transmission-localized surface plasmon resonance. Anal. Chem..

[96] Zhang J., Dai S., Ma C., Di J., Zhao J. (2017). Compact surface plasmon holographic microscopy for near-field film mapping. Opt. Lett..

[97] Zhang Y., Lai J., Yin C., Li Z. (2009). Determination of effective complex refractive index of a turbid liquid with surface plasmon resonance phase detection. Appl. Opt..

[98] Karlsson R. (2004). SPR for molecular interaction analysis: A review of emerging application areas. J. Mol. Recognit..

[99] Lu J., Spasic D., Delport F., Van Stappen T., Detrez I., Daems D., Vermeire S., Gils A., Lammertyn J. (2017). Immunoassay for Detection of Infliximab in Whole Blood Using a Fiber-Optic Surface Plasmon Resonance Biosensor. Anal. Chem..

[100] Vashist S.K., Vashist S.K., Schneider E.M., Luong J.H.T. (2014). Surface plasmon resonance-based immunoassay for human fetuin A. Analyst.

[101] Vashist S.K., Schneider E.M., Luong J.H.T. (2015). Surface plasmon resonance-based immunoassay for human C-reactive protein. Analyst.

[102] Piliarik M., Bocková M., Homola J. (2010). Surface plasmon resonance biosensor for parallelized detection of protein biomarkers in diluted blood plasma. Biosens. Bioelectron..

[103] Vance S.A., Sandros M.G. (2014). Zeptomole detection of C-reactive protein in serum by a nanoparticle amplified surface plasmon resonance imaging aptasensor. Sci. Rep..

[104] Das S., Gupta A., Vaishnavi T.V., Walia S., Bhatia D., Chakraborty B. (2022). Aptamers functionalized biomolecular nano-vehicles for applications in cancer diagnostics & therapeutics. Appl. NanoMedicine.

[105] Hassan E.M., DeRosa M.C. (2020). Recent advances in cancer early detection and diagnosis: Role of nucleic acid based aptasensors. TrAC-Trends Anal. Chem..

[106] Bellassai N., D’Agata R., Jungbluth V., Spoto G. (2019). Surface Plasmon Resonance for Biomarker Detection: Advances in Non-invasive Cancer Diagnosis. Front. Chem..

[107] Kumar V., Kukkar D., Hashemi B., Kim K.H., Deep A. (2019). Advanced Functional Structure-Based Sensing and Imaging Strategies for Cancer Detection: Possibilities, Opportunities, Challenges, and Prospects. Adv. Funct. Mater..

[108] Špringer T., Homola J. (2012). Biofunctionalized gold nanoparticles for SPR-biosensor-based detection of CEA in blood plasma. Anal. Bioanal. Chem..

[109] Špringer T., Chadtová Song X., Ermini M.L., Lamačová J., Homola J. (2017). Functional gold nanoparticles for optical affinity biosensing. Anal. Bioanal. Chem..

[110] Suwansa-ard S., Kanatharana P., Asawatreratanakul P., Wongkittisuksa B., Limsakul C., Thavarungkul P. (2009). Comparison of surface plasmon resonance and capacitive immunosensors for cancer antigen 125 detection in human serum samples. Biosens. Bioelectron..

[111] Wang H., Wang X., Wang J., Fu W., Yao C. (2016). A SPR biosensor based on signal amplification using antibody-QD conjugates for quantitative determination of multiple tumor markers OPEN. Nat. Publ. Gr..

[112] Yi X., Ma J., Guan Y., Chen R., Yang L., Xia X. (2017). The feasibility of using mutation detection in ctDNA to assess tumor dynamics. Int. J. Cancer.

[113] Chen H., Hou Y., Ye Z., Wang H., Koh K., Shen Z., Shu Y. (2014). Label-free surface plasmon resonance cytosensor for breast cancer cell detection based on nano-conjugation of monodisperse magnetic nanoparticle and folic acid. Sens. Actuators B Chem..

[114] Zhu S., Li H., Yang M., Pang S.W. (2018). Label-free detection of live cancer cells and DNA hybridization using 3D multilayered plasmonic biosensor. Nanotechnology.

[115] Wang S.-S., Zhao X.-P., Liu F.-F., Younis M.R., Xia X.-H., Wang C. (2019). Direct Plasmon-Enhanced Electrochemistry for Enabling Ultrasensitive and Label-Free Detection of Circulating Tumor Cells in Blood. Anal. Chem..

[116] Huang X., Hu X., Song S., Mao D., Lee J., Koh K., Zhu Z., Chen H. (2020). Triple-enhanced surface plasmon resonance spectroscopy based on cell membrane and folic acid functionalized gold nanoparticles for dual-selective circulating tumor cell sensing. Sens. Actuators B Chem..

[117] Tadimety A., Zhang Y., Kready K.M., Palinski T.J., Tsongalis G.J., Zhang J.X. (2019). Design of peptide nucleic acid probes on plasmonic gold nanorods for detection of circulating tumor DNA point mutations. Biosens. Bioelectron..

[118] Adams B.D., Anastasiadou E., Esteller M., He L., Slack F.J. (2015). The Inescapable Influence of Noncoding RNAs in Cancer. Cancer Res.

[119] Van der Ven C.F.T., Hogewoning B.C.R., van Mil A., Sluijter J.P.G. (2020). Non-coding RNAs in Cardiac Regeneration. Adv. Exp. Med. Biol..

[120] Zhang L., Wang J., Zhang J., Liu Y., Wu L., Shen J., Zhang Y., Hu Y., Fan Q., Huang W. (2017). Individual Au-Nanocube Based Plasmonic Nanoprobe for Cancer Relevant MicroRNA Biomarker Detection. ACS Sensors.

[121] Ki J.S., Lee H.Y., Son H.Y., Huh Y.-M., Haam S. (2019). Sensitive Plasmonic Detection of miR-10b in Biological Samples Using Enzyme-Assisted Target Recycling and Developed LSPR Probe. ACS Appl. Mater. Interfaces.

[122] Li Q., Wang Q., Yang X., Wang K., Zhang H., Nie W. (2017). High sensitivity surface plasmon resonance biosensor for detection of microRNA and small molecule based on graphene oxide-gold nanoparticles composites. Talanta.

[123] Xue T., Liang W., Li Y., Sun Y., Xiang Y., Zhang Y., Dai Z., Duo Y., Wu L., Qi K. (2019). Ultrasensitive detection of miRNA with an antimonene-based surface plasmon resonance sensor. Nat. Commun..

[124] Mujica M.L., Zhang Y., Bédioui F., Gutiérrez F., Rivas G. (2020). Label-free graphene oxide–based SPR genosensor for the quantification of microRNA21. Anal. Bioanal. Chem..

[125] Fathi F., Rahbarghazi R., Movassaghpour A.A., Rashidi M.-R. (2019). Detection of CD133-marked cancer stem cells by surface plasmon resonance: Its application in leukemia patients. Biochim. Biophys. Acta-Gen. Subj..

[126] Yavas O., Aćimović S.S., Guirado J.G., Berthelot J., Dobosz P., Sanz V., Quidant R. (2018). Self-Calibrating On-Chip Localized Surface Plasmon Resonance Sensing for Quantitative and Multiplexed Detection of Cancer Markers in Human Serum. ACS Sens..

[127] Szymańska B., Lukaszewski Z., Hermanowicz-Szamatowicz K., Gorodkiewicz E. (2020). A biosensor for determination of the circulating biomarker CA125/MUC16 by Surface Plasmon Resonance Imaging. Talanta.

[128] Chiu N.-F., Lin T.-L., Kuo C.-T. (2018). Highly sensitive carboxyl-graphene oxide-based surface plasmon resonance immunosensor for the detection of lung cancer for cytokeratin 19 biomarker in human plasma. Sens. Actuators B Chem..

[129] Karami P., Khoshsafar H., Johari-Ahar M., Arduini F., Afkhami A., Bagheri H. (2019). Colorimetric immunosensor for determination of prostate specific antigen using surface plasmon resonance band of colloidal triangular shape gold nanoparticles. Spectrochim. Acta Part A Mol. Biomol. Spectrosc..

[130] Sankiewicz A., Romanowicz L., Laudanski P., Zelazowska-Rutkowska B., Puzan B., Cylwik B., Gorodkiewicz E. (2016). SPR imaging biosensor for determination of laminin-5 as a potential cancer marker in biological material. Anal. Bioanal. Chem..

[131] Wu W., Wu Q., Ren S.-N., Liu Z., Chen F.-F. (2021). Ti3C2-MXene-assisted signal amplification for sensitive and selective surface plasmon resonance biosensing of biomarker. Chin. J. Anal. Chem..

[132] Raposo G., Stoorvogel W. (2013). Extracellular vesicles: Exosomes, microvesicles, and friends. J. Cell Biol..

[133] Mathivanan S., Ji H., Simpson R.J. (2010). Exosomes: Extracellular organelles important in intercellular communication. J. Proteom..

[134] El Andaloussi S., Mäger I., Breakefield X.O., Wood M.J.A. (2013). Extracellular vesicles: Biology and emerging therapeutic opportunities. Nat. Rev. Drug Discov..

[135] Azmi A.S., Bao B., Sarkar F.H. (2013). Exosomes in cancer development, metastasis, and drug resistance: A comprehensive review. Cancer Metastasis Rev..

[136] Wang Z., Chen J.-Q., Liu J.-L., Tian L. (2016). Exosomes in tumor microenvironment: Novel transporters and biomarkers. J. Transl. Med..

[137] Zhang X., Yuan X., Shi H., Wu L., Qian H., Xu W. (2015). Exosomes in cancer: Small particle, big player. J. Hematol. Oncol..

[138] Wang Q., Zou L., Yang X., Liu X., Nie W., Zheng Y., Cheng Q., Wang K. (2019). Direct quantification of cancerous exosomes via surface plasmon resonance with dual gold nanoparticle-assisted signal amplification. Biosens. Bioelectron..

[139] Mao Z., Zhao J., Chen J., Hu X., Koh K., Chen H. (2021). A simple and direct SPR platform combining three-in-one multifunctional peptides for ultra-sensitive detection of PD-L1 exosomes. Sens. Actuators B Chem..

[140] Thakur A., Qiu G., Ng S.-P., Guan J., Yue J., Lee Y., Wu C.-M.L. (2017). Direct detection of two different tumor-derived extracellular vesicles by SAM-AuNIs LSPR biosensor. Biosens. Bioelectron..

[141] Ibn Sina A.A., Vaidyanathan R., Wuethrich A., Carrascosa L.G., Trau M. (2019). Label-free detection of exosomes using a surface plasmon resonance biosensor. Anal. Bioanal. Chem..

[142] Li J., Ji C., Lü B., Rodin M., Paradies J.H.H., Yin M., Kuckling D. (2020). Dually Crosslinked Supramolecular Hydrogel for Cancer Biomarker Sensing. ACS Appl. Mater. Interfaces.

[143] Thurmond R., Wadsworth S.A., Schafer P.H., Zivin R.A., Siekierka J.J. (2001). Kinetics of small molecule inhibitor binding to p38 kinase. JBIC J. Biol. Inorg. Chem..

[144] Adamczyk M., Moore J.A., Yu Z. (2000). Application of Surface Plasmon Resonance toward Studies of Low-Molecular-Weight Antigen–Antibody Binding Interactions. Methods.

[145] Loo J.F.-C., Yang C., Tsang H.L., Lau P.M., Yong K.-T., Ho H.P., Kong S.K. (2017). An Aptamer Bio-barCode (ABC) assay using SPR, RNase H, and probes with RNA and gold-nanorods for anti-cancer drug screening. Analyst.

[146] Li J., Li Y., Lu F., Liu L., Ji Q., Song K., Yin Q., Lerner R.A., Yang G., Xu H. (2020). A DNA-encoded library for the identification of natural product binders that modulate poly (ADP-ribose) polymerase 1, a validated anti-cancer target. Biochem. Biophys. Res. Commun..

[147] Huang R., Jing X., Huang X., Pan Y., Fang Y., Liang G., Liao Z.-X., Wang H.-S., Chen Z.-F., Zhang Y. (2020). Bifunctional Naphthoquinone Aromatic Amide-Oxime Derivatives Exert Combined Immunotherapeutic and Antitumor Effects through Simultaneous Targeting of Indoleamine-2,3-dioxygenase and Signal Transducer and Activator of Transcription 3. J. Med. Chem..

[148] Wen M., Deng Z.-K., Jiang S.-L., Guan Y.-D., Wu H.-Z., Wang X.-L., Xiao S.-S., Zhang Y., Yang J.-M., Cao D.-S. (2019). Identification of a Novel Bcl-2 Inhibitor by Ligand-Based Screening and Investigation of Its Anti-cancer Effect on Human Breast Cancer Cells. Front. Pharmacol..

[149] Gassner C., Lipsmeier F., Metzger P., Beck H., Schnueriger A., Regula J.T., Moelleken J. (2014). Development and validation of a novel SPR-based assay principle for bispecific molecules. J. Pharm. Biomed. Anal..

[150] Ritter G., Cohen L.S., Williams C.J., Richards E.C., Old L.J., Welt S. (2001). Serological Analysis of Human Anti-Human Antibody Responses in Colon Cancer Patients Treated with Repeated Doses of Humanized Monoclonal Antibody A33. Cancer Res..

[151] Rogozińska-Szczepka J., Utracka-Hutka B., Grzybowska E., Mąka B., Nowicka E., Smok-Ragankiewicz A., Zientek H., Steffen J., Wojciechowska-Łącka A. (2004). BRCA1 and BRCA2 mutations as prognostic factors in bilateral breast cancer patients. Ann. Oncol..

[152] Iovine R., Loscrí V., Pizzi S., Tarparelli R., Vegni A.M. (2013). Model of Multi-Source Nanonetworks for the Detection of BRCA1 DNA Alterations Based on LSPR Phenomenon. Adv. Nanoparticles.

[153] Meenakshi A., Kumar R.S., Kumar N.S. (2002). Elisa for Quantitation of Serum C-erbB-2 Oncoprotein in Breast Cancer Patients. J. Immunoass. Immunochem..

[154] Eletxigerra U., Martinez-Perdiguero J., Barderas R., Pingarrón J.M., Campuzano S., Merino S. (2016). Surface plasmon resonance immunosensor for ErbB2 breast cancer biomarker determination in human serum and raw cancer cell lysates. Anal. Chim. Acta.

[155] Shabani A., Tabrizian M. (2013). Design of a universal biointerface for sensitive, selective, and multiplex detection of biomarkers using surface plasmon resonance imaging. Analyst.

[156] Li R., Feng F., Chen Z.-Z., Bai Y.-F., Guo F.-F., Wu F.-Y., Zhou G. (2015). Sensitive detection of carcinoembryonic antigen using surface plasmon resonance biosensor with gold nanoparticles signal amplification. Talanta.

[157] Lee J.U., Nguyen A.H., Sim S.J. (2015). A nanoplasmonic biosensor for label-free multiplex detection of cancer biomarkers. Biosens. Bioelectron..

[158] Su F., Xu C., Taya M., Murayama K., Shinohara Y., Nishimura S.-I. (2008). Detection of Carcinoembryonic Antigens Using a Surface Plasmon Resonance Biosensor. Sensors.

[159] Altintas Z., Uludag Y., Gurbuz Y., Tothill I.E. (2011). Surface plasmon resonance based immunosensor for the detection of the cancer biomarker carcinoembryonic antigen. Talanta.

[160] Duan F., Zhang S., Yang L., Zhang Z., He L., Wang M. (2018). Bifunctional aptasensor based on novel two-dimensional nanocomposite of MoS2 quantum dots and g-C3N4 nanosheets decorated with chitosan-stabilized Au nanoparticles for selectively detecting prostate specific antigen. Anal. Chim. Acta.

[161] Malic L., Sandros M.G., Tabrizian M. (2011). Designed Biointerface Using Near-Infrared Quantum Dots for Ultrasensitive Surface Plasmon Resonance Imaging Biosensors. Anal. Chem..

[162] Law W.-C., Yong K.-T., Baev A., Prasad P.N. (2011). Sensitivity Improved Surface Plasmon Resonance Biosensor for Cancer Biomarker Detection Based on Plasmonic Enhancement. ACS Nano.

[163] Jung J., Na K., Lee J., Kim K.-W., Hyun J. (2009). Enhanced surface plasmon resonance by Au nanoparticles immobilized on a dielectric SiO_2_ layer on a gold surface. Anal. Chim. Acta.

[164] Huang L., Reekmans G., Saerens D., Friedt J.-M., Frederix F., Francis L., Muyldermans S., Campitelli A., Van Hoof C. (2005). Prostate-specific antigen immunosensing based on mixed self-assembled monolayers, camel antibodies and colloidal gold enhanced sandwich assays. Biosens. Bioelectron..

[165] Jin L.-H., Li S.-M., Cho Y.-H. (2012). Enhanced detection sensitivity of pegylated CdSe/ZnS quantum dots-based prostate cancer biomarkers by surface plasmon-coupled emission. Biosens. Bioelectron..

[166] Acimovic S.S., Ortega M., Sanz V., Berthelot J., Garcia-Cordero J.L., Renger J., Maerkl S.J., Kreuzer M.P., Quidant R. (2014). LSPR Chip for Parallel, Rapid, and Sensitive Detection of Cancer Markers in Serum. Nano Lett..

[167] Vaisocherová H., Šípová H., Víšová I., Bocková M., Špringer T., Ermini M.L., Song X., Krejčík Z., Chrastinová L., Pastva O. (2015). Rapid and sensitive detection of multiple microRNAs in cell lysate by low-fouling surface plasmon resonance biosensor. Biosens. Bioelectron..

[168] Chiu N.-F., Kuo C.-T., Lin T.-L., Chang C.-C., Chen C.-Y. (2017). Ultra-high sensitivity of the non-immunological affinity of graphene oxide-peptide-based surface plasmon resonance biosensors to detect human chorionic gonadotropin. Biosens. Bioelectron..

[169] Chung J., Bernhardt R., Pyun J. (2006). Sequential analysis of multiple analytes using a surface plasmon resonance (SPR) biosensor. J. Immunol. Methods.

[170] Piliarik M., Vaisocherová H., Homola J. (2005). A new surface plasmon resonance sensor for high-throughput screening applications. Biosens. Bioelectron..

[171] Boozer C., Ladd J., Chen S., Jiang S. (2006). DNA-Directed Protein Immobilization for Simultaneous Detection of Multiple Analytes by Surface Plasmon Resonance Biosensor. Anal. Chem..

[172] Chung J., Bernhardt R., Pyun J. (2006). Additive assay of cancer marker CA 19-9 by SPR biosensor. Sens. Actuators B Chem..

[173] Chang C.-C., Chiu N.-F., Lin D.S., Chu-Su Y., Liang Y.-H., Lin C.-W. (2010). High-Sensitivity Detection of Carbohydrate Antigen 15-3 Using a Gold/Zinc Oxide Thin Film Surface Plasmon Resonance-Based Biosensor. Anal. Chem..

[174] Liang Y.-H., Chang C.-C., Chen C.-C., Chu-Su Y., Lin C.-W. (2012). Development of an Au/ZnO thin film surface plasmon resonance-based biosensor immunoassay for the detection of carbohydrate antigen 15-3 in human saliva. Clin. Biochem..

[175] Kim H.-M., Jeong D.H., Lee H.-Y., Park J.-H., Lee S.-K. (2021). Design and validation of fiber optic localized surface plasmon resonance sensor for thyroglobulin immunoassay with high sensitivity and rapid detection. Sci. Rep..

[176] Melo S.A., Luecke L.B., Kahlert C., Fernandez A.F., Gammon S.T., Kaye J., LeBleu V.S., Mittendorf E.A., Weitz J., Rahbari N. (2015). Glypican-1 identifies cancer exosomes and detects early pancreatic cancer. Nature.

[177] Xiong H., Huang Z., Lin Q., Yang B., Yan F., Liu B., Chen H., Kong J. (2022). Surface Plasmon Coupling Electrochemiluminescence Immunosensor Based on Polymer Dots and AuNPs for Ultrasensitive Detection of Pancreatic Cancer Exosomes. Anal. Chem..

[178] Raghu D., Christodoulides J.A., Christophersen M., Liu J.L., Anderson G.P., Robitaille M., Byers J.M., Raphael M.P. (2018). Nanoplasmonic pillars engineered for single exosome detection. PLoS ONE.

[179] Ahmed N., Barker G., Oliva K.T., Hoffmann P., Riley C., Reeve S., Smith I., E Kemp B., A Quinn M., E Rice G. (2004). Proteomic-based identification of haptoglobin-1 precursor as a novel circulating biomarker of ovarian cancer. Br. J. Cancer.

[180] Bharti A., Ma P.C., Maulik G., Singh R., Khan E., Skarin A.T., Salgia R. (2004). Haptoglobin α-Subunit and Hepatocyte Growth Factor Can Potentially Serve as Serum Tumor Biomarkers in Small Cell Lung Cancer. Anticancer Res..

[181] Xing P.X., Young G., Ho D., Sinatra M.A., Hoj P.B., McKenzie I.F.C. (1996). A new approach to fecal occult blood testing based on the detection of haptoglobin. Cancer.

[182] Bresalier R.S., Byrd J.C., Tessler D., Lebel J., Koomen J., Hawke D., Half E., Liu K.-F., Mazurek N. (2004). A circulating ligand for galectin-3 is a haptoglobin-related glycoprotein elevated in individuals with colon cancer. Gastroenterology.

[183] Kazuno S., Fujimura T., Arai T., Ueno T., Nagao K., Fujime M., Murayama K. (2011). Multi-sequential surface plasmon resonance analysis of haptoglobin–lectin complex in sera of patients with malignant and benign prostate diseases. Anal. Biochem..

[184] Lleonart M.E., Cajal S.R.Y., Groopman J.D., Friesen M.D. (2004). Sensitive and specific detection of K-ras mutations in colon tumors by short oligonucleotide mass analysis. Nucleic Acids Res..

[185] D’Agata R., Bellassai N., Allegretti M., Rozzi A., Korom S., Manicardi A., Melucci E., Pescarmona E., Corradini R., Giacomini P. (2020). Direct plasmonic detection of circulating RAS mutated DNA in colorectal cancer patients. Biosens. Bioelectron..

[186] Sutphen R., Xu Y., Wilbanks G.D., Fiorica J., Grendys J.E.C., LaPolla J.P., Arango H., Hoffman M.S., Martino M., Wakeley K. (2004). Lysophospholipids Are Potential Biomarkers of Ovarian Cancer. Cancer Epidemiol. Biomark. Prev..

[187] Yuan J., Duan R., Yang H., Luo X., Xi M. (2012). Detection of serum human epididymis secretory protein 4 in patients with ovarian cancer using a label-free biosensor based on localized surface plasmon resonance. Int. J. Nanomed..

[188] Wu A., Wu B., Guo J., Luo W., Wu D., Yang H., Zhen Y., Yu X., Wang H., Zhou Y. (2011). Elevated expression of CDK4 in lung cancer. J. Transl. Med..

[189] Sankiewicz A., Lukaszewski Z., Trojanowska K., Gorodkiewicz E. (2016). Determination of collagen type IV by Surface Plasmon Resonance Imaging using a specific biosensor. Anal. Biochem..

[190] Zhou Y., Liao Q., Han Y., Chen J., Liu Z., Ling H., Zhang J., Yang W., Oyang L., Xia L. (2016). Rac1 overexpression is correlated with epithelial mesenchymal transition and predicts poor prognosis in non-small cell lung cancer. J. Cancer.

[191] Sahu V., Gupta A., Kumar R., Gupta T., Mohan A., Dey S. (2016). Quantification of Rac1 and Rac1b in serum of non small cell lung cancer by label free real time assay. Clin. Chim. Acta.

[192] Cennamo N., Pesavento M., Lunelli L., Vanzetti L., Pederzolli C., Zeni L., Pasquardini L. (2015). An easy way to realize SPR aptasensor: A multimode plastic optical fiber platform for cancer biomarkers detection. Talanta.

[193] Cho H., Yeh E.-C., Sinha R., Laurence T.A., Bearinger J.P., Lee L.P. (2012). Single-Step Nanoplasmonic VEGF_165_Aptasensor for Early Cancer Diagnosis. ACS Nano.

[194] Yang C.-Y., Brooks E., Li Y., Denny P., Ho C.-M., Qi F., Shi W., Wolinsky L., Wu B., Wong D.T.W. (2005). Detection of picomolar levels of interleukin-8 in human saliva by SPR. Lab Chip.

[195] Lin Y.-T., Darvishi S., Preet A., Huang T.-Y., Lin S.-H., Girault H.H., Wang L., Lin T.-E. (2020). A Review: Electrochemical Biosensors for Oral Cancer. Chemosensors.

[196] Vergara D., Bianco M., Pagano R., Priore P., Lunetti P., Guerra F., Bettini S., Carallo S., Zizzari A., Pitotti E. (2018). An SPR based immunoassay for the sensitive detection of the soluble epithelial marker E-cadherin. Nanomed. Nanotechnol. Biol. Med..

[197] Zhou W., Ma Y., Yang H., Ding Y., Luo X. (2011). A label-free biosensor based on silver nanoparticles array for clinical detection of serum p53 in head and neck squamous cell carcinoma. Int. J. Nanomed..

[198] Myers M.J., Smith E.R., Turfle P.G. (2017). Biomarkers in Veterinary Medicine. Annu. Rev. Anim. Biosci..

[199] Loyez M., Albert J., Caucheteur C., Wattiez R. (2018). Cytokeratins Biosensing Using Tilted Fiber Gratings. Biosensors.

[200] Aubé A., Charbonneau D.M., Pelletier J.N., Masson J.-F. (2016). Response Monitoring of Acute Lymphoblastic Leukemia Patients Undergoing l-Asparaginase Therapy: Successes and Challenges Associated with Clinical Sample Analysis in Plasmonic Sensing. ACS Sens..

[201] Krupin O., Wang C., Berini P. (2015). Detection of leukemia markers using long-range surface plasmon waveguides functionalized with Protein G. Lab Chip.

[202] Zhao Y., Tong L., Li Y., Pan H., Zhang W., Guan M., Li W., Chen Y., Li Q., Li Z. (2016). Lactose-Functionalized Gold Nanorods for Sensitive and Rapid Serological Diagnosis of Cancer. ACS Appl. Mater. Interfaces.

[203] Lee K.-A., Ahn J.-Y., Lee S.-H., Sekhon S.S., Kim D.-G., Min J., Kim Y.-H. (2015). Aptamer-based Sandwich Assay and its Clinical Outlooks for Detecting Lipocalin-2 in Hepatocellular Carcinoma (HCC). Sci. Rep..

[204] Gorodkiewicz E., Charkiewicz R., Rakowska A., Bajko P., Chyczewski L., Niklinski J. (2012). SPR imaging biosensor for podoplanin: Sensor development and application to biological materials. Microchim. Acta.

[205] Gill K., Mohanti B.K., Ashraf S., Singh A.K., Dey S. (2012). Quantification of p38αMAP kinase: A prognostic marker in HNSCC with respect to radiation therapy. Clin. Chim. Acta.

[206] Gorodkiewicz E., Sieńczyk M., Regulska E., Grzywa R., Pietrusewicz E., Lesner A., Łukaszewski Z. (2012). Surface plasmon resonance imaging biosensor for cathepsin G based on a potent inhibitor: Development and applications. Anal. Biochem..

[207] Hsieh H.-Y., Chang R., Huang Y.-Y., Juan P.-H., Tahara H., Lee K.-Y., Vo D.N.K., Tsai M.-H., Wei P.-K., Sheen H.-J. (2022). Continuous polymerase chain reaction microfluidics integrated with a gold-capped nanoslit sensing chip for Epstein-Barr virus detection. Biosens. Bioelectron..

